# Ten‐year clinical outcomes after drug‐eluting stents implantation according to clinical presentation—Insights from the DECADE cooperation

**DOI:** 10.1111/eci.14323

**Published:** 2024-10-01

**Authors:** Fabian Starnecker, J. J. Coughlan, Lisette Okkels Jensen, Sarah Bär, Sebastian Kufner, Salvatore Brugaletta, Lorenz Räber, Michael Maeng, Luis Ortega‐Paz, Dik Heg, Karl‐Ludwig Laugwitz, Manel Sabaté, Stephan Windecker, Adnan Kastrati, Kevin Kris Warnakula Olesen, Salvatore Cassese

**Affiliations:** ^1^ Klinik für Herz‐ und Kreislauferkrankungen, Deutsches Herzzentrum München Technische Universität München Munich Germany; ^2^ DZHK (German Center for Cardiovascular Research), partner site Munich Heart Alliance Munich Germany; ^3^ Cardiovascular Research Institute, Mater Private Network Dublin Ireland; ^4^ Department of Cardiology Odense University Hospital Odense Denmark; ^5^ Department of Cardiology Inselspital, Bern University Hospital, University of Bern Bern Switzerland; ^6^ Hospital Clinic, Institut d'Investigacions Biomèdiques August Pi i Sunyer, University of Barcelona Barcelona Spain; ^7^ Department of Cardiology Aarhus University Hospital Aarhus Denmark; ^8^ Division of Cardiology University of Florida College of Medicine Jacksonville Florida USA; ^9^ Clinical Trials Unit Bern University of Bern Bern Switzerland; ^10^ Medizinische Klinik und Poliklinik, Klinikum rechts der Isar Technische Universität München Munich Germany; ^11^ Centro de Investigación Biomédica en Red de Enfermedades Cardiovasculares (CIBERCV), CIBERCV CB16/11/00411 Madrid Spain

**Keywords:** acute coronary syndrome, chronic coronary syndrome, drug‐eluting stents, NSTE‐ACS, percutaneous coronary intervention, STEMI

## Abstract

**Background:**

Investigations of very long‐term outcomes after percutaneous coronary intervention (PCI) with drug‐eluting stents (DES) according to clinical presentation are scarce. Here, we investigated the 10‐year clinical outcomes of patients undergoing DES‐PCI according to clinical presentation.

**Methods:**

Patient‐level data from five randomized trials with 10‐year follow‐up after DES‐PCI were pooled. Patients were dichotomized into acute coronary syndrome (ACS) or chronic coronary syndrome (CCS) groups as per clinical presentation. The primary outcome was all‐cause death. Secondary outcomes were cardiovascular death, myocardial infarction (MI), definite stent thrombosis (ST) and repeat revascularization involving the target lesion (TLR), target vessel (TVR) or non‐target vessel (nTVR).

**Results:**

Of the 9700 patients included in this analysis, 4557 presented with ACS and 5143 with CCS. Compared with CCS patients, ACS patients had a higher risk of all‐cause death and nTVR in the first year, but comparable risk thereafter. In addition, ACS patients had a higher risk of MI [adjusted hazard ratio 1.21, 95% confidence interval (1.04–1.41)] and definite ST [adjusted hazard ratio 1.48, 95% confidence interval (1.14–1.92)], while the risk of TLR and TVR was not significantly different up to 10‐year follow‐up.

**Conclusions:**

Compared to CCS patients, ACS patients treated with PCI and DES implantation have an increased risk of all‐cause death and repeat revascularization of remote vessels up to 1 year, with no significant differences thereafter and up to 10‐year follow‐up. ACS patients have a consistently higher risk of MI and definite ST. Whether these differences persist with current antithrombotic and secondary prevention therapies requires further investigation.

## Graphical abstract

Long‐term outcomes of patients undergoing percutaneous coronary intervention with drug‐eluting stents for acute coronary syndrome (ACS) or chronic coronary syndrome (CCS) were investigated. Compared with CCS patients, ACS patients have an increased risk of all‐cause death and repeat revascularization of remote vessels up to 1‐year follow‐up, with no significant differences thereafter. ACS patients have a consistently higher risk of MI and definite ST up to 10‐year follow‐up.

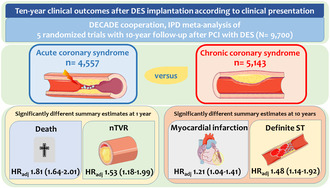

**Key Points**

1. In the first year after PCI with DES, the risk of all‐cause death and repeat revascularization of remote vessels is higher in ACS patients than in CCS patients. After the first year, the risk is not significantly different between the two groups up to 10‐year follow‐up.
2. Compared to CCS patients, ACS patients are at increased risk for MI and definite ST through to 10 years after PCI with DES.
3. The risk of TLR and TVR does not differ significantly between ACS and CCS patients through to 10‐year follow‐up after PCI with DES.

## 1 INTRODUCTION

It has been reported that clinical outcomes in patients undergoing percutaneous coronary intervention (PCI) may vary according to clinical presentation.^1^, ^2^, ^3^, ^4^ Previous studies have reported increased short‐term mortality in patients treated for acute coronary syndrome (ACS) compared to patients treated for chronic coronary syndrome (CCS).^2^, ^5^ ACS patients have also been reported to have an increased risk of early (0–30 days) stent thrombosis (ST).^3^, ^6^, ^7^

However, while previous studies have evaluated outcomes through to 5 years after PCI, analyses at longer follow‐up remain limited.^1^, ^8^ The few longer‐term analyses that exist are based on study cohorts recruited more than two decades ago, in which patients were treated with bare metal stent (BMS) or angioplasty alone, being only a minority treated with drug‐eluting stent (DES) platforms.^1^, ^8^ Given that, over the past 20 years, DES platforms have seen a continuous iteration that has led to unprecedented levels of safety and efficacy, even in complex clinical settings,^9^ it is less certain whether the differences in clinical outcomes according to clinical presentation persist at very long‐term follow‐up in the DES era.

For this reason, in this analysis, we examined clinical outcomes according to clinical presentation in PCI patients treated with DES in randomized trials with a follow‐up of 10 years.

## 2 METHODS

### 2.1 Study population

The DECADE cooperation (Adverse Events and Coronary Artery Disease Progression) is an analysis of individual patient data from five randomized controlled studies with 10‐year follow‐up. All PCI patients pooled in this dataset were treated with DES. Details and objective of the DECADE cooperation as well as the inclusion and exclusion criteria of the trials included have been described previously.^10^, ^11^ Briefly, the DECADE cooperation is an investigator‐initiated scientific cooperation including data from the EXAMINATION (Clinical Evaluation of the Xience‐V Stent in Acute Myocardial Infarction),^12^ the ISAR‐TEST 4 (Intracoronary Stenting and Angiographic Results: Test Efficacy of three Limus‐Eluting Stents),^13^ the ISAR‐TEST 5 (Intracoronary Stenting and Angiographic Results: Test Efficacy of Sirolimus‐ and Probucol‐Eluting Versus Zotarolimus‐Eluting Stents),^14^ the SORT OUT III (Randomized Clinical Comparison of the Endeavour and the Cypher Coronary Stents in Non‐Selected Angina Pectoris Patients),^15^ and the SIRTAX (Sirolimus‐Eluting vs. Paclitaxel‐Eluting Stents for Coronary Revascularization) trials.^16^

For the current analysis, PCI patients treated with DES implantation were stratified into two groups (ACS vs. CCS) as per their clinical presentation at time of index procedure. The classification into the ACS and CCS groups was according to the definitions in the original trials. Key inclusion and exclusion criteria and the primary outcomes of each trial included in the present analysis, as well as peri‐ and post‐procedural medications have been published previously.^10^, ^11^ Patients from the EXAMINATION trial that were treated with BMS were excluded from the present analysis.^12^ Data on ST from the SORT OUT III trial were limited to a 5‐year follow‐up.^17^ All five trials were approved by the ethics committee or the institutional review board at the study sites and written informed patient consent was obtained before study inclusion. The 10‐year results of each of the five trials have been published previously.^17^, ^18^, ^19^, ^20^, ^21^ The data that support the findings of this study are available from the principal investigators of the individual randomized trials upon reasonable request.

### 2.2 Outcomes

The primary outcome of the current analysis was all‐cause death. Secondary outcomes included cardiovascular death, MI, definite ST, target‐lesion revascularization (TLR), target‐vessel revascularization (TVR) and non‐target‐vessel revascularization (nTVR). All outcomes were investigated according to the original study definitions, as published elsewhere.^10^, ^11^

### 2.3 Statistical analysis

The analysis of clinical data was performed at patient‐level using a 1‐stage approach by entering a cluster effect by parent study in all univariable and multivariable models, with a focus on clinical presentation. The analysis of angiographic and procedural data was at lesion level. Continuous variables are provided as means ±SD or medians with 25th–75th percentiles. Categorical data are presented as counts or proportions (%). The ANOVA test (continuous data) and the *χ* ^2^ or Fisher exact test where the expected cell value was <5 (categorical variables) were used to check for significance of differences between the groups. A two‐tailed *p*‐value <.05 was considered statistically significant. Event‐free survival was analysed using the Kaplan–Meier method. The log‐rank test was used to detect differences between the groups. Hazard ratios before and after adjustment for baseline imbalances (HR, HR_adj_) and 95% CIs were estimated using a Cox proportional hazards model. The proportional hazards assumption was checked by the Grambsch and Thernau method.^22^ Fulfilment of the proportional hazards assumption was assessed according to the weighted residuals and by checking the graph of the scaled Schoenfeld residuals.^22^ For outcomes other than all‐cause death and cardiovascular death, cumulative incidence functions accounting for competing risks were calculated with the *cmprsk* package in R (based on the model by Fine and Grey) and compared by a Cox proportional hazards model.^23^, ^24^ Conventional multivariable analyses were performed after adjustment for the age (represented as a continuous variable without any transformation), body mass index, sex, diabetes mellitus, DES‐generation (early vs. newer‐generation DES), hypertension, smoking and hypercholesterolemia, history of MI, multivessel disease and vessel treated with clustering for trial. The rationale for selecting these factor variables was based on knowledge of their correlation with clinical outcomes.^25^ A potential impact of between‐study heterogeneity on outcomes related to clinical presentation was investigated by adding an interaction term between study and clinical presentation and between study arm and clinical presentation in all multivariable models. We performed adjusted landmark analyses for all outcomes of interest with a landmark at 1 year. For MI and definite ST we selected an additional landmark at 30 days. Potential interactions between clinical presentation and age (≥75 years vs. <75 years), sex, diabetes mellitus and DES‐generation were examined for all outcomes of interest by entering an interaction term in the adjusted Cox proportional hazards model and calculating a *p‐*value for interaction (*p*_int_). An additional subgroup analysis was performed according to the type of ACS at clinical presentation (non‐ST elevation ACS [NSTE‐ACS] and ST‐elevation myocardial infarction [STEMI]). In addition, we calculated the summary estimates for all outcomes of interest according to the presence of single or multivessel CAD. All analyses were performed using the R 3.6.0 Statistical Package (R Foundation for Statistical Computing, Vienna, Austria).

## 3 RESULTS

This analysis included 9700 patients undergoing PCI with DES implantation. These patients were divided into two groups by clinical presentation: 4557 patients with ACS and 5143 patients with CCS. Overall, 738 patients (7.6%) had a follow‐up shorter than 9.5 years. The median follow‐up duration in this latter group was 5.3 years [4.6; 6.8].

### 3.1 Baseline characteristics

The patient‐level baseline characteristics are displayed in Table 1. ACS patients were younger with a lower cardiovascular risk, less frequent previous MI and multivessel CAD as compared with CCS patients. However, smoking was more frequent in patients presenting with ACS. ACS patients received more often newer‐generation DES platforms. Table 2 presents the lesion‐level baseline angiographic and procedural characteristics. Interestingly, minimal lumen diameter was smaller among ACS patients before PCI, and smaller among CCS patients after PCI.

**TABLE 1:** Patient‐level baseline characteristics by clinical presentation.

Characteristics	ACS (*n* = 4557)	CCS (*n* = 5143)	*p*‐value
Age	64.5 (12.1)	66.5 (10.4)	<.001
Women, *n* (%)	1114 (24.4)	1182 (23.0)	.095
BMI	27.3 (4.5)	27.6 (4.4)	.006
Ejection fraction (%)	51.2 (11.7)	55.2 (11.5)	<.001
Diabetes, *n* (%)	1017 (22.3)	1281 (24.9)	.003
Insulin‐dependent	296 (6.5)	342 (6.7)	.791
Hypertension, *n* (%)	2449 (54.4)	3474 (68.1)	<.001
Smoke, *n* (%)	1466 (32.8)	899 (17.8)	<.001
Hypercholesterolemia, *n* (%)	2501 (55.5)	3609 (70.7)	<.001
Previous MI	970 (21.6)	1577 (30.9)	<.001
No. of diseased coronary vessels			
1 vessel	1373 (39.2)	900 (23.3)	<.001
2 vessels	840 (24.0)	958 (24.8)	
3 vessels	1292 (36.9)	2005 (51.9)	
No. of lesions	1.33 (.63)	1.38 (.64)	<.001
Trials, *n* (%)			
EXAMINATION	751 (16.5)	–	<.001
ISAR‐TEST 4	1060 (23.3)	1543 (30.0)	
ISAR‐TEST 5	1232 (27.0)	1770 (34.4)	
SIRTAX	462 (10.1)	550 (10.7)	
SORT OUT III	1052 (23.1)	1280 (24.9)	
DES newer‐generation, *n* (%)	3299 (72.4)	3567 (69.4)	.001
DES Type, *n* (%)			
Yukon Choice BP‐SES	541 (11.9)	758 (14.7)	<.001
Cypher PP‐SES	1025 (22.5)	1300 (25.3)	
Endeavour PP‐ZES	506 (11.1)	656 (12.8)	
ISAR VIVO/Coroflex PF SPES	811 (17.8)	1191 (23.2)	
Resolute PP‐ZES	421 (9.2)	579 (11.3)	
Taxus PP‐PES	233 (5.1)	276 (5.4)	
Xience PP‐EES	1020 (22.4)	383 (7.5)	

*Note*: Data are mean ± SD or counts (%). Data were analysed at a patient level. Completeness of data: ejection fraction was not available in 3296 patients (1569 in the ACS group and 1727 in the CCS group); BMI was not available in 212 patients (131 in the ACS group and 81 in the CCS group); hypertension status was not available in 91 patients (53 in the ACS group and 38 in the CCS group); diabetic status was not available in 1 patient in the ACS group; hypercholesterolemia status was not available in 87 patients (48 in the ACS group and 39 in the CCS group); number of diseased coronary vessels was not available in 2332 patients (1052 in the ACS group and 1280 in the CCS group); previous myocardial infarction status was not available in 102 patients (56 in the ACS group and 46 in the CCS group). The remaining data are complete.

Abbreviations: ACS, acute coronary syndrome; BMI, body mass index; CCS, chronic coronary syndrome; EXAMINATION, Clinical Evaluation of the Xience‐V Stent in Acute Myocardial Infarction; ISAR‐TEST 4, Intracoronary Stenting and Angiographic Results: Test Efficacy of 3 Limus‐Eluting Stents; ISAR‐TEST 5, Intracoronary Stenting and Angiographic Results: Test Efficacy of Sirolimus‐ and Probucol‐Eluting Versus Zotarolimus‐Eluting Stents; SIRTAX, Sirolimus‐Eluting Versus Paclitaxel‐Eluting Stents for Coronary Revascularization; SORT OUT III, Randomized Clinical Comparison of the Endeavour and the Cypher Coronary Stents in Non‐Selected Angina Pectoris Patients.

**TABLE 2:** Lesion‐level angiographic and procedural characteristics by clinical presentation.

Characteristics	ACS (*n* = 6056)	CCS (*n* = 7120)	*p*‐value
Target vessel			
LM	30 (.5)	50 (.7)	<.001
LAD	2695 (44.5)	3096 (43.5)	
LCX	1389 (22.9)	1845 (25.9)	
RCA	1929 (31.9)	2094 (29.4)	
Venous bypass graft	13 (.2)	35 (.5)	
Bifurcation involved and treated	828/3852 (21.5)	1317/5320 (24.8)	<.001
Complex lesion (type 2B/C)	3585/5280 (67.9)	4219/7063 (59.7)	<.001
	**(*n* = 6033)**	**(*n* = 7080)**	
Pre‐procedural reference vessel diameter, mm	2.8 [2.5; 3.1]	2.8 [2.4; 3.1]	.003
Pre‐procedural minimal lumen diameter, mm	.8 [.4; 1.1]	.9 [.7; 1.3]	<.001
Balloon diameter, mm	3.0 [2.8; 3.5]	3.0 [2.7; 3.5]	.002
Maximal balloon pressure, atm	15.0 [12.0; 17.0]	16.0 [13.0; 18.0]	<.001
Total stented length, mm	22.0 [18.0; 28.0]	18.0 [15.0; 28.0]	<.001
No. of stents	1.00 [1.0; 2.0]	1.00 [1.0; 2.0]	.139
Post‐procedural minimal lumen diameter, mm	2.6 [2.3; 2.9]	2.5 [2.2; 2.9]	.048
Post‐procedural diameter stenosis, %	10.7 [7.1; 14.8]	10.7 [7.4; 14.9]	.274

*Note*: Data are median (interquartile range) or counts (%). Data were analysed at a lesion level. Completeness of data: Preprocedural reference vessel and minimal lumen diameter were not available for 4103 patients (2251 in the ACS group and 1852 in the CCS group); balloon diameter was not available for 159 lesions (71 in the ACS group and 88 in the CCS group); maximal balloon pressure was not available for 4526 lesions (2437 in the ACS group and 2089 in the CCS group); total stented length was not available for 67 lesions (25 in the ACS group and 42 in the CCS group); number of stents was not available for 417 lesions (231 in the ACS group and 186 in the CCS group); and postprocedural minimal lumen diameter and diameter stenosis were not available for 4865 lesions (2584 in the ACS group and 2281 in the CCS group). The remaining data are complete.

Abbreviations: ACS, acute coronary syndrome; CCS chronic coronary syndrome.

### 3.2 Clinical outcomes

Table 3 displays the cumulative incidences of outcomes of interest: because of the non‐fulfilment of the proportional hazards assumption for all‐cause death, cardiovascular death and nTVR, we did not report overall risk estimates for these outcomes for the complete follow‐up of 10 years. The results of Schoenfelds global goodness‐of‐fit test for outcomes of interest are shown in Table S1.

**TABLE 3:** Clinical outcomes through to 10 years by clinical presentation.

Outcome	ACS (*n* = 4557)	CCS (*n* = 5143)	HR (95% CI)	*p‐*value	HR_adj_ (95% CI)	*p‐*value
All‐cause death^a^	1262 (28.8)	1507 (30.7)				
0–1 year	203/4557 (4.5)	148/5143 (2.9)	1.57 (1.19–2.07)	<.01	1.81 (1.64–2.01)	<.001
1–10 years	1059/4338 (25.5)	1359/4968 (28.7)	.87 (.72–1.05)	.16	.97 (.92–1.03)	.35
Cardiovascular death^a^	675 (15.4)	744 (15.2)				
0–1 year	124/4557 (2.7)	74/5143 (1.4)	1.91 (1.32–2.77)	<.001	2.31 (1.78–3.00)	<.001
1–10 years	551/4338 (13.3)	670/4968 (14.2)	.92 (.79–1.07)	.27	.99 (.93–1.06)	.83
MI	374 (8.5)	378 (7.6)	1.11 (.92–1.34)	.27	1.21 (1.04–1.41)	.01
Definite ST	88 (2.0)	72 (1.5)	1.39 (1.15–1.67)	<.001	1.48 (1.14–1.92)	<.01
TLR	622 (13.9)	779 (15.4)	.90 (.69–1.17)	.43	1.05 (.86–1.27)	.63
TVR	845 (18.8)	980 (19.3)	.98 (.76–1.26)	.89	1.14 (.92–1.42)	.22
nTVR^a^	778 (17.3)	1022 (20.3)				
0–1 year	335/4557 (7.4)	300/5143 (5.9)	1.30 (.98–1.72)	.07	1.53 (1.18–1.99)	<.01
1–10 years	443/4013 (11.0)	722/4675 (15.5)	.68 (.51–.92)	.01	.82 (.64–1.06)	.14

*Note*: The numbers shown in brackets are Kaplan–Meier estimates (%). Cumulative incidence functions were computed for outcomes other than death to account for competing risks. The adjusted hazard ratios, 95% CI, and *p‐*values reported here are derived from a conventional multivariable analysis with adjustment for the following variables: age, BMI, sex, diabetes, drug‐eluting stent generation, hypertension, smoking, hypercholesterolemia, history of MI, multivessel disease and vessel treated, with clustering for trial.

Abbreviations: ACS, acute coronary syndrome; CCS chronic coronary syndrome; HR hazard ratio; HR_adj_, adjusted hazard ratio; MI, myocardial infarction; nTVR, nontarget vessel revascularization; ST, stent thrombosis; TLR, target lesion revascularization; TVR, target vessel revascularization.

^a^ Because of the non‐fulfilment of the proportional hazards assumption for all‐cause death, cardiovascular death, and nTVR over 10 years of follow‐up, we refrained from showing overall statistical testing results. Instead, we show the incidences and risk estimates for 0–1 year, and 1–10 years, separately. Note that the cumulative incidences from the separate periods may not sum up to the overall incidence.

**FIGURE 1:**
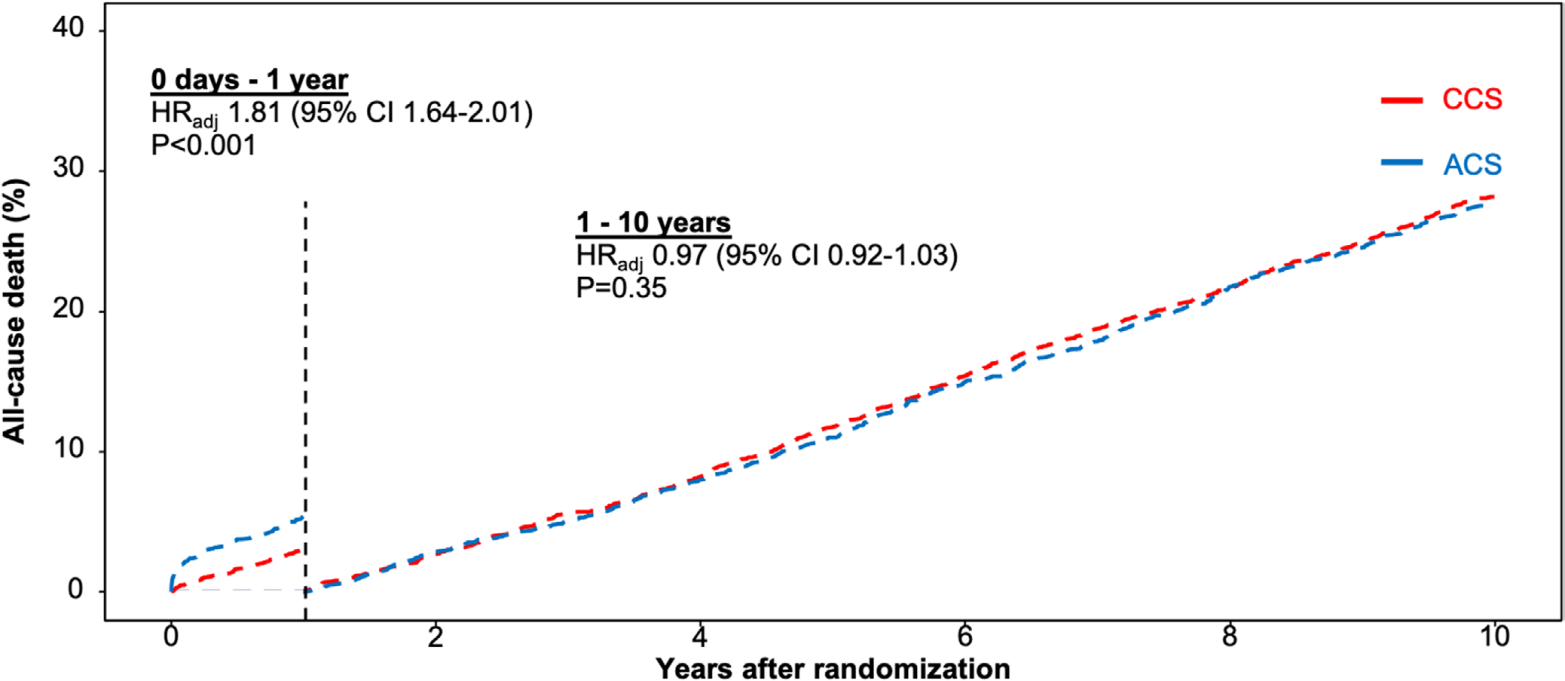
Landmark analysis of all‐cause death by clinical presentation. Adjusted landmark analysis of all‐cause death by clinical presentation from 0 days to 1 year and from 1 to 10 years. ACS indicates acute coronary syndrome; CCS, chronic coronary syndrome.

All‐cause death occurred in 1262 ACS patients (27.7%) and in 1507 CCS patients (29.3%). From 0 days through to 1 year after PCI with DES, all‐cause death occurred in 203 of 4557 ACS patients and in 148 of 5143 CCS patients (4.5% vs. 2.9%; HR_adj_ 1.81 [95% CI, 1.64–2.01]; *p* < .001). From 1 to 10 years, all‐cause death occurred in 1059 of 4338 ACS patients and in 1359 of 4968 CCS patients (25.5% vs. 28.7%, HR_adj_ .97 [95% CI, .92–1.03]; *p* = .35; Figure 1).

Cardiovascular death occurred in 675 ACS patients (14.8%) and in 744 CCS patients (14.5%). From 0 days through to 1 year after PCI with DES, cardiovascular death occurred in 124 of 4557 ACS patients in 74 of 5143 CCS patients (2.7% vs. 1.4%, HR_adj_ 2.31 [95% CI, 1.78–3.00]; *p* < .001). From 1 to 10 years, cardiovascular death occurred in 551 of 4338 ACS patients and in 670 of 4968 CCS patients (13.3% vs. 14.2%, HR_adj_ .99 [95%CI, .93–1.06]; *p* = .83; Figure S3).

**FIGURE 2:**
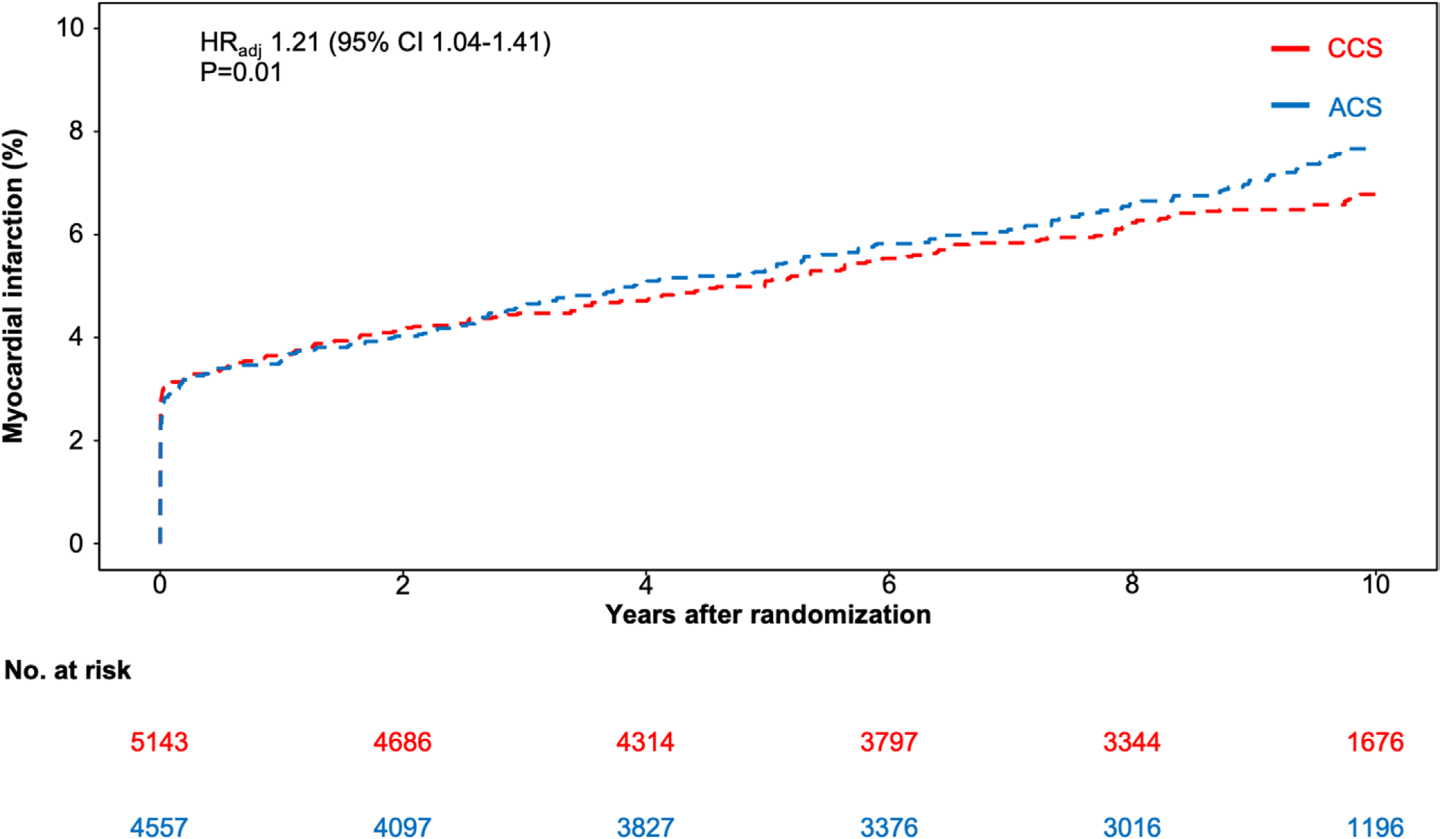
Ten‐year cumulative incidence of myocardial infarction by clinical presentation. Adjusted cumulative incidence function curves and adjusted hazard ratio (HR_adj_) with accompanying 95% CI for myocardial infarction by clinical presentation. ACS indicates acute coronary syndrome; CCS, chronic coronary syndrome.

MI occurred in 374 ACS patients (8.2%) and in 378 CCS patients (7.3%). There was a significantly higher risk of MI in ACS patients as compared with CCS patients (HR_adj_ 1.21 [95% CI, 1.04–1.41]; *p* = .01; Figure 2).

**FIGURE 3:**
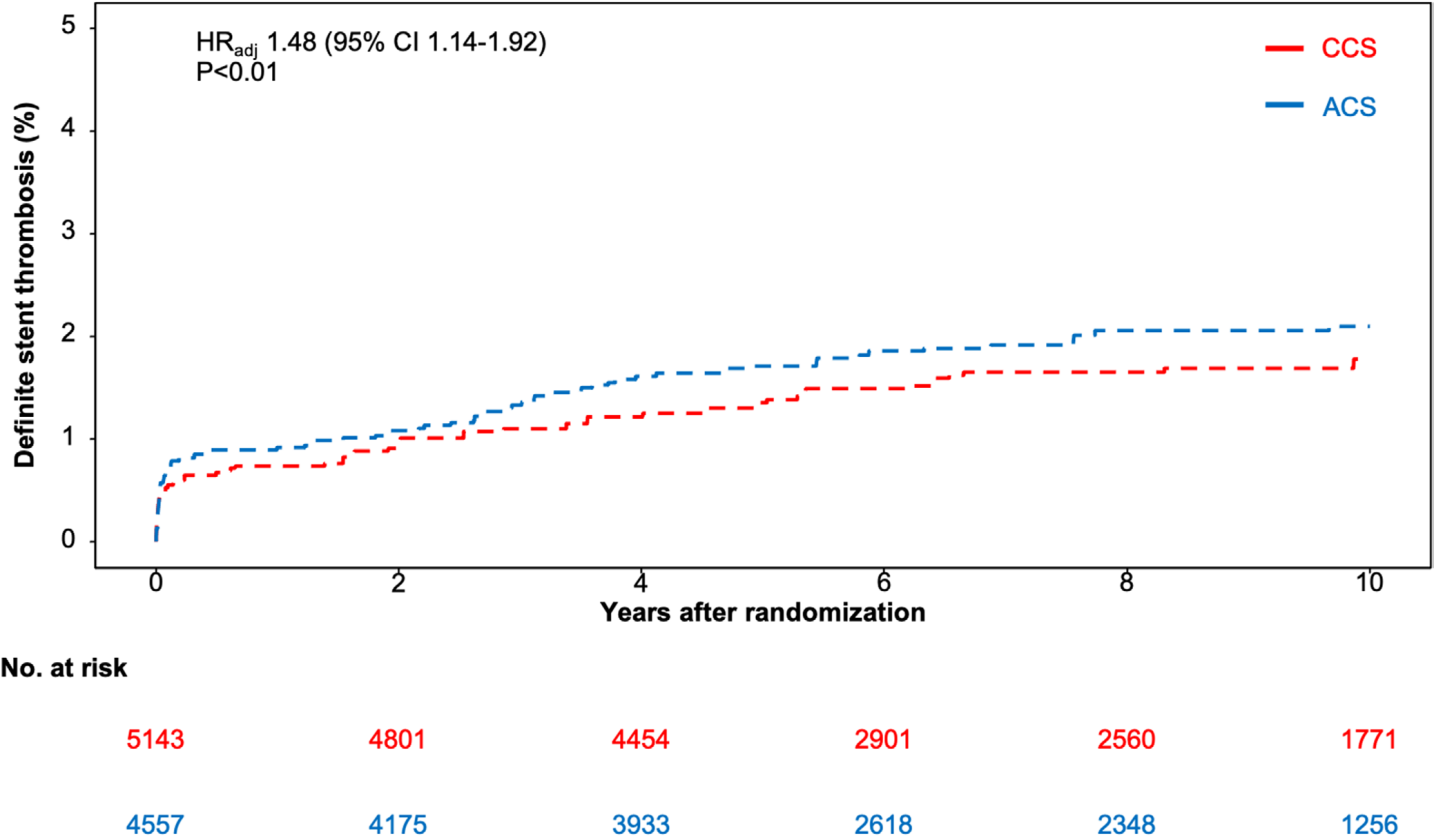
Ten‐year cumulative incidence of definite stent thrombosis by clinical presentation. Adjusted cumulative incidence function curves and adjusted hazard ratio (HR_adj_) with accompanying 95% CI for definite stent thrombosis by clinical presentation. ACS indicates acute coronary syndrome; CCS, chronic coronary syndrome.

Definite ST occurred in 88 ACS patients (1.9%) and in 72 CCS patients (1.4%). There was a significantly higher risk of definite ST in ACS patients as compared with CCS patients (HR_adj_ 1.48 [95% CI, 1.14–1.92]; *p* < .01; Figure 3).

TLR occurred in 622 ACS patients (13.6%) and in 779 CCS patients (15.1%). There was no significant difference in terms of TLR between groups (HR_adj_ 1.05 [95% CI, .86–1.27]; *p* = .63; Figure S1).

TVR occurred in 845 ACS patients (18.5%) and in 980 CCS patients (19.1%). There was no significant difference in terms of TVR between groups (HR_adj_ 1.14 [95% CI, .92–1.42]; *p* = .22; Figure S2).

nTVR occurred in 778 ACS patients (17.1%) and in 1022 CCS patients (19.9%). From 0 days through to 1 year after PCI with DES, nTVR occurred in 335 of 4557 ACS patients and in 300 of 5143 CCS patients (7.4% vs. 5.9%, HR_adj_ 1.53 [95% CI, 1.18–1.99]; *p* < .01). From 1 to 10 years, nTVR occurred in 443 of 4013 ACS patients and in 722 of 4675 individuals (11.0% vs. 15.5%; HR_adj_ .82 [95% CI, .64–1.06]; *p* = .14; Figure S8).

### 3.3 Landmark analyses

The results of the landmark analysis for MI, definite ST, TLR and TVR as per clinical presentation are reported in the Appendix (Table S2 and Figures S4–S7). Of interest, as compared to CCS patients, ACS patients displayed a significantly higher risk for MI (HR_adj_ 1.43 [95% CI, 1.23–1.66]; *p* < .001) and a marginally higher risk for definite ST (HR_adj_ 1.48 [95%CI, 1.00–2.21]; *p* = .05) beyond the first year after PCI with DES.

### 3.4 Effect of age, sex, diabetes mellitus and DES generation

The results of the subgroup analyses are shown in the Figures S9–S15. Of interest, there was a significant interaction between clinical presentation and age with respect to TVR and nTVR. There was also a significant interaction between clinical presentation and sex with respect to all‐cause death. A significant interaction between clinical presentation and diabetes was identified with respect to all‐cause death, cardiovascular death, MI and TVR. No significant interaction was found between clinical presentation and DES generation for any of the outcomes of interest.

### 3.5 Clinical outcomes according to ACS type and CAD burden

The baseline characteristics and clinical outcomes of ACS patients according to the type of ACS at clinical presentation are reported in Tables S3–S5. Of interest, the risk of all‐cause death was not significantly different between NSTE‐ACS and STEMI patients. The outcomes according to number of diseased coronary vessels at baseline are shown in Table S6.

## 4 DISCUSSION

The present analysis evaluated 10‐year outcomes of nearly 10,000 ACS and CCS patients undergoing PCI with DES implantation. The main findings are as follows:

1. In the first year, the risk of all‐cause death and repeat revascularization of remote vessels is higher in ACS patients than in CCS patients. After the first year, the risk for these outcomes is not significantly different between the two groups.
2. Compared to CCS patients, ACS patients are at increased risk for MI and definite ST through to 10 years.
3. The risk of TLR and TVR does not differ significantly between ACS and CCS patients through to 10‐year follow‐up.

On the one hand, previous studies on this topic were mostly limited by the sample size, the follow‐up duration and the fact that patients were not treated exclusively with DES.^1^, ^26^, ^27^ On the other hand, the significant improvement in DES technology, including the evolution towards platforms with ultra‐thin struts, has been shown to reduce the short‐ and mid‐term risk of target lesion related ischemic events.^28^, ^29^ Yet, a recent meta‐analysis revealed that the reduction in ischemic events up to 1 year associated with contemporary DES platforms was consistent among ACS and CCS patients.^9^ In this respect, the present study represents an important addition to previous knowledge on this research topic.

We found that clinical presentation with ACS was associated with increased mortality within the first year after PCI with DES. After 1 year, mortality rates were comparable between ACS and CCS patients. These results support previous observations that the mortality rate in patients treated for ACS is higher in the first period after PCI and highlight the need for aggressive secondary prevention as early as possible after ACS.^1^, ^2^, ^5^, ^30^, ^31^ Notably, women with ACS were at higher risk of death as compared to men. Although the exploratory nature of the subgroup analysis makes it impossible to determine the exact causality of this result, this difference is probably due to the fact that women tend to develop and present any manifestation of CAD at a later age than men.^11^

In the first year after PCI with DES, ACS presentation was associated with a higher risk of nTVR than CCS. After 1 year, nTVR rates were comparable between the two groups. The concept of ACS as a systemic disease affecting the entire coronary tree beyond the culprit coronary vessel has been confirmed by several imaging studies showing the unstable nature of lesions outside the culprit vessel with an increased risk of plaque rupture and subsequent thrombotic events.^32^, ^33^ This finding also supports the concept of complete revascularization in ACS, as recommended by the 2023 European Society of Cardiology guidelines for the management of ACS.^30^ However, despite differences in the risk of repeat revascularization over time in patients with ACS and CCS, secondary prevention goals do not distinguish between ACS and CCS and do not take into account the variability of this risk over time.^30^, ^34^ Based on current results, a more aggressive secondary prevention in the early stages after ACS (namely, the first year), a period in which the risk of recurrent events appears to be higher, might be reasonable. In this sense, the role of intravascular imaging in ACS patients to assess the extent and the nature of non‐culprit lesions is likely to increase in the coming years, as data suggest that almost half of subsequent thrombotic events in this context are related to progression of atherosclerotic disease in remote coronary segments or vessels.^35^, ^36^

ACS patients were more likely to experience recurrent MI up to 10 years after PCI with DES implantation. Although ACS has been identified as predictive for recurrent MI,^37^ we did not find a significant difference in terms of MI between ACS patients and CCS patients within 30 days after PCI. The presence of positive cardiac biomarkers at baseline could potentially mask a significant number or recurrent MI after PCI in the early phase of intervention (detection bias). Conversely, we found an increased risk of recurrent MI in ACS patients compared to CCS patients from 1 to 10 years after PCI, albeit this was not associated with increased mortality. The lack of surrogacy between recurrent MI and mortality over the long term is not novel^38^ and probably reflects the complex interplay between diagnostic sensitivity, heterogeneity of myocardial damage and improvement in background medical therapy, which has probably contributed to a weakening of the causal relationship between MI and mortality.

Patients with ACS at time of PCI with DES were at increased risk of definite ST through to 10 years, though the rates of early and late definite ST were not significant different between groups. Of note, we found no significant interaction according to DES generation. We also found no difference in ST rates according to the number of diseased coronary vessels at baseline. Previous analyses reported an increased risk of early ST and a comparable risk of late ST in patients with ACS compared to patients with CCS.^6^, ^39^ In the current study, the majority of definite ST events occurred within the first 30 days after PCI in both groups, highlighting the need for more potent platelet inhibition in the early phase, regardless of clinical presentation.^6^, ^39^, ^40^ Interestingly, 1 year after PCI, the rates of definite ST were numerically higher among ACS patients, even this difference was not statistically significant. Of note, in the analysis of ACS subtypes, NSTE‐ACS patients had a lower risk of definite ST compared to STEMI patients. This is consistent with previous analyses reporting that STEMI presentation is associated with the highest risk of ST among CAD subsets.^6^, ^41^, ^42^

### 4.1 Study limitations

The current analysis has several limitations. Firstly, there were significant differences between the ACS and CCS groups in terms of baseline features. Although the statistical adjustment sought to account for baseline confounders, residual bias due to unmeasured factors cannot be definitively ruled out. Secondly, we did not have data on whether nTVR procedures were due to staged PCI, new ACS events or were supported by the presence of inducible cardiac ischemia. In fact, this information was not routinely captured in the electronic case report forms of the original trials included in this analysis. However, the persistently higher risk of MI beyond 1 year in the ACS group compared to the CCS group confirms the higher disease burden of the ACS group. Thirdly, due to the long‐term follow‐up accumulated, our analysis included data from cohorts recruited several years ago. For this reason, the current findings may not reflect contemporary practice. Among others, whether the differences observed in our cohort in patients treated predominantly with clopidogrel also occur in patients treated with more effective antiplatelet agents cannot be explored in this context. Finally, there was no information concerning adherence to pharmacological treatment, so we were unable to determine the impact of premature discontinuation of antiplatelet therapy or lipid‐lowering drugs on the outcomes of interest.

## 5 CONCLUSIONS

In patients undergoing PCI with DES, clinical presentation with ACS compared to CCS is associated with a higher risk of all‐cause death and nTVR up to 1 year, with no significant differences thereafter and up to 10‐year follow‐up. Patients undergoing PCI with DES for ACS have an increased risk of ST and recurrent MI that persists through to 10‐year follow‐up. Whether these differences are maintained with current antithrombotic and secondary prevention therapies requires further investigation.

## AUTHOR CONTRIBUTIONS

F.S. was principally responsible for drafting the article. J.J.C. assisted with drafting the manuscript. A.K. was primarily responsible for the statistical analysis and provided critical feedback on the first draft. S.C. was primarily responsible for conception of the project and provided critical feedback on the first draft. A.K. and S.C. had direct access to and can verify the data reported in the article. M. Maeng, L.R., S. Bär, A.A., L.O.J., S. Brugaletta, L.O.‐P., K.‐L.L., D.H., M.S., S.K., K.K.W.O., and S.W. all provided editorial revision and feedback to the first draft and worked on the databases of the original trials. All authors reviewed the submitted version of the manuscript prior to submission.

## CONFLICT OF INTEREST STATEMENT

Dr. Brugaletta reports advisory board fees from Boston Scientific and lectures fee from Abbott Vascular. Dr. Kufner reports lecture fees from AstraZeneca, Bristol Myers Squib, and Translumina. Dr. Windecker reports research and educational grants to the institution from Abbott, Abiomed, Amgen, AstraZeneca, Bayer, Biotronik, Boehringer Ingelheim, Boston Scientific, Bristol Myers Squibb, Cardinal Health, CardioValve, Corflow Therapeutics, CSL Behring, Daiichi Sankyo, Edwards Lifesciences, Guerbet, InfraRedx, Janssen‐Cilag, Johnson & Johnson, Medicure, Medtronic, Merck Sharp & Dohm, Miracor Medical, Novartis, Novo Nordisk, Organon, OrPha Suisse, Pfizer, Polares, Regeneron, Sanofi‐Aventis, Servier, Sinomed, Terumo, Vifor, and V‐Wave. Dr. Windecker serves as unpaid advisory board member and/or unpaid member of the steering/executive group of trials funded by Abbott, Abiomed, Amgen, AstraZeneca, Bayer, Boston Scientific, Biotronik, Bristol Myers Squibb, Edwards Lifesciences, Janssen, MedAlliance, Medtronic, Novartis, Polares, Recardio, Sinomed, Terumo, V‐Wave, and Xeltis, but he has not received personal payments by pharmaceutical companies or device manufacturers. Dr. Windecker is also a member of the steering/executive committee group of several investigator‐initiated trials that receive funding by industry without an effect on his personal remuneration. Dr. Maeng is supported by a grant from the Novo Nordisk Foundation (grant number NNF22OC0074083), has received lecture and/or advisory board fees from Astra‐Zeneca, Bayer, Boehringer‐Ingelheim, Bristol‐Myers Squibb, and Novo Nordisk, has received research grants from Philips, Bayer and Novo Nordisk, has received a travel grant from Novo Nordisk, has ongoing institutional research contracts with Janssen, Novo Nordisk and Philips, and is a minor stockholder in Novo Nordisk, Eli Lilly & Company, and Verve Therapeutics. The other authors report no conflicts.

## Supporting information


Data S1.
