# Supplementary material for: Ten‐year clinical outcomes after drug‐eluting stents implantation according to clinical presentation—Insights from the DECADE cooperation
Source: Eur J Clin Invest. 2024 Oct 1;55(1):e14323. doi: 10.1111/eci.14323 (PMC11628648; doi:10.1111/eci.14323)

**APPENDIX**

**Supplemental Tables**

- **Table S1. Schoenfelds global goodness-of-fit test for outcomes of interest**
- **Table S2. Landmark analysis for selected secondary outcomes by clinical presentation**
- **Table S3. Baseline characteristics by type of acute coronary syndrome**
- **Table S4. Angiographic and procedural characteristics by type of acute coronary syndrome**
- **Table S5. Clinical outcomes by type of acute coronary syndrome**
- **Table S6. Clinical outcomes by number of diseased coronary vessels**

**Supplemental Figures**

- **Figure S1. Ten-year cumulative incidence of target lesion revascularization by clinical presentation**
- **Figure S2. Ten-year cumulative incidence of target vessel revascularization by clinical presentation**
- **Figure S3. Landmark analysis of cardiovascular death by clinical presentation**
- **Figure S4. Landmark analysis of myocardial infarction by clinical presentation**
- **Figure S5. Landmark analysis of definite stent thrombosis by clinical presentation**
- **Figure S6. Landmark analysis of target lesion revascularization by clinical presentation**
- **Figure S7. Landmark analysis of target vessel revascularization by clinical presentation**
- **Figure S8. Landmark analysis of non-target vessel revascularization by clinical presentation**
- **Figure S9. Subgroup analysis of all-cause death by clinical presentation**
- **Figure S10. Subgroup analysis of cardiovascular death by clinical presentation**
- **Figure S11. Subgroup analysis of myocardial infarction by clinical presentation**
- **Figure S12. Subgroup analysis of definite stent thrombosis by clinical presentation**
- **Figure S13. Subgroup analysis of target lesion revascularization by clinical presentation**
- **Figure S14. Subgroup analysis of target vessel revascularization by clinical presentation**
- **Figure S15. Subgroup analysis of non-target vessel revascularization by clinical presentation**

**SUPPLEMENTAL TABLES**

**Table S1. Schoenfelds global goodness-of-fit test for outcomes of interest**

| Outcome | P value |
| --- | --- |
| All-cause death | <0.01 |
| Cardiovascular death | <0.001 |
| Myocardial infarction | 0.08 |
| Definite stent thrombosis | 0.38 |
| Target Lesion Revascularization | 0.35 |
| Target Vessel Revascularization | 0.06 |
| Non-Target Vessel Revascularization | <0.001 |

**Table S2. Landmark analysis for selected secondary outcomes by clinical presentation**

| Outcome | ACS  (n=4,557) | CCS  (n=5,143) | HR (95% CI) | P value | HR_adj_ (95% CI) | P value |
| --- | --- | --- | --- | --- | --- | --- |
| Myocardial infarction |  |  |  |  |  |  |
| *0-30 days* | 103 (2.3) | 124 (2.4) | 0.94 (0.64-1.36) | 0.735 | 1.00 (0.77-1.31) | 0.973 |
| *30 days-1 year* | 28/4,385 (0.6) | 29/4,987 (0.6) | 1.10 (0.67-1.81) | 0.700 | 1.32 (0.67-2.59) | 0.415 |
| *1-10 years* | 243/4,233 (5.9) | 225/4,838 (4.8) | 1.21 (0.99-1.48) | 0.061 | 1.43 (1.23-1.66) | <0.001 |
| Definite stent thrombosis |  |  |  |  |  |  |
| *0-30 days* | 30 (0.7) | 20 (0.4) | 1.70 (1.12-2.61) | 0.013 | 1.46 (0.98-2.16) | 0.059 |
| *30 days-1 year* | 12/4,452 (0.3) | 12/5,089 (0.2) | 1.15 (0.85-1.55) | 0.377 | 1.37 (0.80-2.35) | 0.248 |
| *1-10 years* | 46/4,305 (1.2) | 40/4,941 (0.9) | 1.30 (0.88-1.92) | 0.181 | 1.48 (1.00-2.21) | 0.051 |
| TLR |  |  |  |  |  |  |
| *0-1 year* | 309 (6.8) | 384 (7.5) | 0.92 (0.67-1.25) | 0.590 | 1.08 (0.82-1.42) | 0.572 |
| *1-10 years* | 313/4,044 (7.8) | 395/4,596 (8.7) | 0.88 (0.70-1.12) | 0.301 | 1.02 (0.86-1.21) | 0.815 |
| TVR |  |  |  |  |  |  |
| *0-1 year* | 475/4,556 (10.4) | 539 (10.5) | 1.01 (0.73-1.41) | 0.945 | 1.21 (0.90-1.63) | 0.204 |
| *1-10 years* | 370/3,884 (9.4) | 441/4,443 (9.9) | 0.95 (0.79-1.14) | 0.551 | 1.05 (0.91-1.22) | 0.470 |

Data are counts (Kaplan-Meier estimates in percent); ACS indicates acute coronary syndrome; CCS chronic coronary syndrome; CI: confidence interval; HR: hazard ratio (unadjusted); HRadj: hazard ratio (adjusted); TLR: target lesion revascularization; TVR: target vessel revascularization

**Table S3. Patient-level baseline characteristics by type of acute coronary syndrome**

| Characteristics | NSTE-ACS  (n=2,791) | STEMI  (n=1,766) | *P* value |
| --- | --- | --- | --- |
| Age | 66.2 (11.5) | 61.8 (12.5) | <0.001 |
| Women, n (%) | 749 (26.8) | 365 (20.7) | <0.001 |
| BMI | 27.4 (4.7) | 27.2 (4.1) | 0.072 |
| Ejection fraction (%) | 53.4 (11.7) | 48.5 (11.1) | <0.001 |
| Diabetics, n (%) | 686 (24.6) | 331 (18.8) | <0.001 |
| *Insulin-dependent* | 232 (8.3) | 64 (3.6) | <0.001 |
| Hypertension, n (%) | 1,691 (61.5) | 758 (43.2) | <0.001 |
| Smoker, n (%) | 677 (24.9) | 789 (45.1) | <0.001 |
| Hypercholesterolemia, n (%) | 1,763 (64.0) | 738 (42.0) | <0.001 |
| Previous MI | 806 (29.4) | 164 (9.3) | <0.001 |
| No. of diseased coronary vessels |  |  | <0.001 |
| *1 vessel* | 429 (22.5) | 939 (58.8) |  |
| *2 vessels* | 486 (25.5) | 354 (22.2) |  |
| *3 vessels* | 991 (51.9) | 301 (18.9) |  |
| No. of lesions | 1.44 (0.7) | 1.17 (0.4) | <0.001 |
| Trials, n (%) |  |  | <0.001 |
| *EXAMINATION* | 0 (0.0) | 751 (42.5) |  |
| *ISAR-TEST 4* | 753 (27.0) | 307 (17.4) |  |
| *ISAR-TEST 5* | 921 (33.0) | 311 (17.6) |  |
| *SIRTAX* | 235 (8.4) | 227 (12.9) |  |
| *SORT OUT III* | 882 (31.6) | 170 (9.6) |  |
| DES newer-generation, n (%) | 1,930 (69.2) | 1,369 (77.5) | <0.001 |
| DES Type, n (%) |  |  | <0.001 |
| *Yukon Choice BP-SES* | 374 (13.4) | 167 (9.5) |  |
| *Cypher PP-SES* | 738 (26.4) | 287 (16.3) |  |
| *Endeavor PP-ZES* | 436 (15.6) | 70 (4.0) |  |
| *ISAR VIVO/Coroflex PF SPES* | 596 (21.4) | 215 (12.2) |  |
| *Resolute PP-ZES* | 325 (11.6) | 96 (5.4) |  |
| *Taxus PP-PES* | 123 (4.4) | 110 (6.2) |  |
| *Xience PP-EES* | 199 (7.1) | 821 (46.5) |  |

NSTE-ACS indicates non-ST-segment elevation acute coronary syndrome; STEMI ST-segment elevation myocardial infarction.

EXAMINATION, Clinical Evaluation of the Xience-V Stent in Acute Myocardial Infarction; ISAR-TEST 4, Intracoronary Stenting and Angiographic Results: Test Efficacy of 3 Limus-Eluting Stents; ISAR-TEST 5, Intracoronary Stenting and Angiographic Results: Test Efficacy of Sirolimus- and Probucol-Eluting Versus Zotarolimus-Eluting Stents; SIRTAX, Sirolimus-Eluting Versus Paclitaxel-Eluting Stents for Coronary Revascularization; and SORT OUT III, Randomized Clinical Comparison of the Endeavor and the Cypher Coronary Stents in Non-Selected Angina Pectoris Patients.

Data are mean ±SD or counts (%). Data were analyzed at a patient level. Completeness of data: Ejection fraction was not available in 1,569 patients (440 in the STEMI group and 1,129 in the NSTE-ACS group); BMI was not available in 131 patients (73 in the STEMI group and 58 in the NSTE-ACS group); hypertension status was not available in 53 patients (11 in the STEMI group and 42 in the NSTE-ACS group); diabetic status was not available in 1 patient in the STEMI group; hypercholesterolemia status was not available in 48 patients (10 in the STEMI group and 38 in the NSTE-ACS group); number of diseased coronary vessels was not available in 1,052 patients (170 in the STEMI group and 882 in the NSTE-ACS group); previous myocardial infarction status was not available in 56 patients (11 in the STEMI group and 45 in the NSTE-ACS group). The remaining data are complete.

**Table S4. Lesion-level angiographic and procedural characteristics by type of acute coronary syndrome**

|  | NSTE-ACS  (n=4,010) | STEMI  (n=2,046) | P value |
| --- | --- | --- | --- |
| Target vessel |  |  | <0.001 |
| *LM* | 26 (0.7) | 4 (0.2) |  |
| *LAD* | 1,794 (44.7) | 901 (44.0) |  |
| *LCX* | 1,017 (25.4) | 372 (18.2) |  |
| *RCA* | 1162 (29.0) | 767 (37.5) |  |
| *Venous bypass graft* | 11 (0.3) | 2 (0.1) |  |
| Bifurcation involved and treated | 664/2,754 (24.1) | 164/1,098 (14.9) | <0.001 |
| Complex lesion (type 2B/C) | 2,625/3,990 (65.8) | 960/1,290 (74.4) | <0.001 |
|  | **(n=3,992)** | **(n=2,041)** |  |
| Pre-procedural reference vessel diameter, mm | 2.8 [2.4; 3.1] | 2.8 [2.5; 3.2] | <0.001 |
| Pre-procedural minimal lumen diameter, mm | 0.9 [0.6; 1.2] | 0.4 [0.0; 0.9] | <0.001 |
| Balloon diameter, mm | 3.1 [2.7; 3.5] | 3.0 [2.8; 3.5] | 0.025 |
| Maximal balloon pressure, atm | 15.0 [12.2; 17.0] | 14.0 [12.0; 16.0] | <0.001 |
| Total stented length, mm | 18.0 [16.0; 28.0] | 23.0 [18.0; 32.0] | <0.001 |
| No. of stents | 1.00 [1.0; 2.0] | 1.00 [1.0; 2.0] | 0.016 |
| Post-procedural minimal lumen diameter, mm | 2.6 [2.2; 2.9] | 2.6 [2.3; 2.9] | 0.001 |
| Post-procedural diameter stenosis, % | 10.8 [7.1; 14.9] | 10.6 [7.0; 14.8] | 0.438 |

NSTE-ACS indicates non-ST-segment elevation acute coronary syndrome; STEMI ST-segment elevation myocardial infarction.

Data are median (interquartile range) or counts (%). Data were analyzed at a lesion level. Completeness of continuous data: Preprocedural reference vessel and minimal lumen diameter were not available for 2251 patients (973 in the STEMI group and 1,278 in the NSTE-ACS group); balloon diameter was not available for 71 lesions (20 in the STEMI group and 51 in the NSTE-ACS group); maximal balloon pressure was not available for 2437 lesions (1,048 in the STEMI group and 1389 in the NSTE-ACS group); total stented length was not available for 25 lesions (6 in the STEMI group and 19 in the NSTE-ACS group); number of stents was not available for 231 lesions (67 in the STEMI group and 164 in the NSTE-ACS group); and postprocedural minimal lumen diameter and diameter stenosis were not available for 2584 lesions (1,113 in the STEMI group and 1,471 in the NSTE-ACS group). The remaining data are complete.

**Table S5. Clinical outcomes by type of acute coronary syndrome**

| Outcomes | NSTE-ACS  (n=2,791) | STEMI  (n=1,766) | HR (95% CI) | P value | HR_adj_ (95% CI) | P value |
| --- | --- | --- | --- | --- | --- | --- |
| All cause death | 867 (32.2) | 395 (23.3) | 1.46 (1.16-1.84) | <0.01 | 0.91 (0.78-1.07) | 0.27 |
| Cardiovascular death | 435 (16.2) | 240 (14.1) | 1.21 (0.72-2.02) | 0.47 | 0.86 (0.58-1.27) | 0.45 |
| Myocardial infarction | 258 (9.5) | 116 (6.8) | 1.48 (1.15-1.91) | <0.01 | 1.05 (0.87-1.26 | 0.62 |
| Definite stent thrombosis | 48 (1.8) | 40 (2.4) | 0.80 (0.49-1.31) | 0.37 | 0.56 (0.39-0.82) | <0.01 |
| Target lesion revascularization | 430 (15.6) | 192 (11.1) | 1.48 (0.86-2.56) | 0.15 | 1.40 (0.96-2.04) | 0.08 |
| Target vessel revascularization | 569 (20.6) | 276 (15.8) | 1.36 (0.85-2.18) | 0.20 | 1.25 (0.92-1.70) | 0.16 |
| Non target vessel revascularization | 533 (19.4) | 245 (14.1) | 1.44 (0.68-3.06) | 0.35 | 1.31 (0.59-2.91) | 0.51 |

NSTE-ACS indicates non-ST elevation acute coronary syndrome; STEMI ST-elevation myocardial infarction; HR hazard ratio; HR_adj_, adjusted hazard ratio; MI, myocardial infarction; nTVR, nontarget vessel revascularization; ST, stent thrombosis; TLR, target lesion revascularization; and TVR, target vessel revascularization. The numbers shown in brackets are Kaplan-Meier estimates (%). Cumulative incidence functions were computed for outcomes other than death to account for competing risks. The adjusted hazard ratios, 95% CI, and *P* values reported here are derived from a conventional multivariable analysis with adjustment for the following variables: age, BMI, sex, diabetes, drug-eluting stent generation, hypertension, smoking, hypercholesterolemia, history of MI, multivessel disease, and vessel treated, with clustering for trial.

**Table S6. Clinical outcomes by number of diseased coronary vessels**

|  | 1-vessel disease (n=2,273) | 2-vessel disease (n=1,798) | 3-vessel disease (n=3,297) | 3-vessel disease vs. 1-vessel disease | | 2-vessel disease vs. 1-vessel disease | | 3-vessel disease vs. 2-vessel disease | |
| --- | --- | --- | --- | --- | --- | --- | --- | --- | --- |
| Outcome |  |  |  | **HR_adj_ (95% CI)** | **P value** | **HR_adj_ (95% CI)** | **P value** | **HR_adj_ (95% CI)** | **P value** |
| All cause death | 478 (22.4) | 466 (27.3) | 1,178 (37.6) | 1.46 (1.33-1.60) | <0.000 | 1.12 (1.04-1.20) | <0.01 | 1.30 (1.24-1.37) | <0.001 |
| Cardiovascular death | 289 (13.6) | 298 (17.7) | 710 (22.9) | 1.43 (1.25-1.64) | <0.001 | 1.16 (1.01-1.34) | 0.04 | 1.23 (1.18-1.29) | <0.001 |
| MI | 129  (5.9) | 84  (4.8) | 252  (7.8) | 1.80 (1.48-2.18) | <0.001 | 0.98 (0.84-1.14) | 0.879 | 1.84 (1.53-2.20) | <0.001 |
| Definite ST | 56  (2.5) | 23  (1.3) | 42  (1.3) | 1.07 (0.65-1.77) | 0.78 | 0.87 (0.75-1.01) | 0.06 | 1.24 (0.78-1.97) | 0.37 |
| TLR | 290 (13.1) | 275 (15.7) | 675 (21.0) | 2.02 (1.44-2.84) | <0.001 | 1.38 (1.07-1.78) | 0.01 | 1.46 (1.33-1.60) | <0.001 |
| TVR | 379 (17.0) | 353 (20.0) | 849 (26.2) | 1.93 (1.36-2.72) | <0.001 | 1.34 (1.03-1.73) | 0.03 | 1.44 (1.30-1.59) | <0.001 |
| nTVR | 289 (13.1) | 383 (21.8) | 1,128 (35.0) | 3.45 (2.31-5.15) | <0.001 | 1.87 (1.17-3.00) | <0.01 | 1.84 (1.52-2.23) | <0.001 |

HR_adj_, adjusted hazard ratio; MI, myocardial infarction; nTVR, non-target vessel revascularization; ST, stent thrombosis; TLR, target lesion revascularization; and TVR, target vessel revascularization. The numbers shown in brackets are Kaplan-Meier estimates (%). Cumulative incidence functions were computed for outcomes other than death to account for competing risks. The adjusted hazard ratios, 95% CI, and *P* values reported here are derived from a conventional multivariable analysis with adjustment for the following variables: age, BMI, sex, diabetes, drug-eluting stent generation, hypertension, smoking, hypercholesterolemia, history of MI, multivessel disease, and vessel treated, with clustering for trial.

**SUPPLEMENTAL FIGURE LEGENDS**

**Figure S1. Ten-year cumulative incidence of target lesion revascularization by clinical presentation.**

Adjusted cumulative incidence function curves and adjusted hazard ratio (HR_adj_) with accompanying 95% CI for target lesion revascularization by clinical presentation. ACS indicates acute coronary syndrome; CCS, chronic coronary syndrome.

**Figure S2. Ten-year cumulative incidence of target vessel revascularization by clinical presentation.**

Adjusted cumulative incidence function curves and adjusted hazard ratio (HR_adj_) with accompanying 95% CI for target vessel revascularization by clinical presentation. ACS indicates acute coronary syndrome; CCS, chronic coronary syndrome.

**Figure S3. Landmark analysis of cardiovascular death by clinical presentation.**

Adjusted landmark analysis of cardiovascular death by clinical presentation from 0 days to 1 year and from 1 to 10 years. ACS indicates acute coronary syndrome; CCS, chronic coronary syndrome.

**Figure S4. Landmark analysis of myocardial infarction by clinical presentation.**

Adjusted landmark analysis of myocardial infarction by clinical presentation from 0 to 30 days, 30 days to 1 year and from 1 to 10 years. ACS indicates acute coronary syndrome; CCS, chronic coronary syndrome.

**Figure S5. Landmark analysis of definite stent thrombosis by clinical presentation.**

Adjusted landmark analysis of definite stent thrombosis by clinical presentation from 0 to 30 days, 30 days to 1 year and from 1 to 10 years. ACS indicates acute coronary syndrome; CCS, chronic coronary syndrome.

**Figure S6. Landmark analysis of target lesion revascularization by clinical presentation.**

Adjusted landmark analysis of target lesion revascularization by clinical presentation from 0 days to 1 year and from 1 to 10 years. ACS indicates acute coronary syndrome; CCS, chronic coronary syndrome.

**Figure S7. Landmark analysis of target vessel revascularization by clinical presentation.**

Adjusted landmark analysis of target vessel revascularization by clinical presentation from 0 days to 1 year and from 1 to 10 years. ACS indicates acute coronary syndrome; CCS, chronic coronary syndrome.

**Figure S8. Landmark analysis of non-target vessel revascularization by clinical presentation.**

Adjusted landmark analysis of non-target vessel revascularization by clinical presentation from 0 days to 1 year and from 1 to 10 years. ACS indicates acute coronary syndrome; CCS, chronic coronary syndrome.

**Figure S9.** **Subgroup analysis of all-cause death by clinical presentation**.

10-year cumulative incidence of all-cause death in ACS versus CCS across subgroups based on age, sex, diabetes and stent generation.

ACS indicates acute coronary syndrome; CCS, chronic coronary syndrome; CI, confidence interval; DES2G, newer-generation DES; HR, hazard ratio; pint, p value for interaction.

**Figure S10.** **Subgroup analysis of cardiovascular death by clinical presentation**.

10-year cumulative incidence of cardiovascular death in ACS versus CCS across subgroups based on age, sex, diabetes and stent generation.

ACS indicates acute coronary syndrome; CCS, chronic coronary syndrome; CI, confidence interval; DES2G, newer-generation DES; HR, hazard ratio; pint, p value for interaction.

**Figure S11.** **Subgroup analysis of myocardial infarction by clinical presentation**.

10-year cumulative incidence of myocardial infarction in ACS versus CCS across subgroups based on age, sex, diabetes and stent generation.

ACS indicates acute coronary syndrome; CCS, chronic coronary syndrome; CI, confidence interval; DES2G, newer-generation DES; HR, hazard ratio; pint, p value for interaction.

**Figure S12.** **Subgroup analysis of definite stent thrombosis by clinical presentation**.

10-year cumulative incidence of definite stent thrombosis in ACS versus CCS across subgroups based on age, sex, diabetes and stent generation.

ACS indicates acute coronary syndrome; CCS, chronic coronary syndrome; CI, confidence interval; DES2G, newer-generation DES; HR, hazard ratio; pint, p value for interaction.

**Figure S13.** **Subgroup analysis of target lesion revascularization by clinical presentation**.

10-year cumulative incidence of target lesion revascularization in ACS versus CCS across subgroups based on age, sex, diabetes and stent generation.

ACS indicates acute coronary syndrome; CCS, chronic coronary syndrome; CI, confidence interval; DES2G, newer-generation DES; HR, hazard ratio; pint, p value for interaction.

**Figure S14.** **Subgroup analysis of target vessel revascularization by clinical presentation**.

10-year cumulative incidence of target vessel revascularization in ACS versus CCS across subgroups based on age, sex, diabetes and stent generation.

ACS indicates acute coronary syndrome; CCS, chronic coronary syndrome; CI, confidence interval; DES2G, newer-generation DES; HR, hazard ratio; pint, p value for interaction.

**Figure S15.** **Subgroup analysis of non-target vessel revascularization by clinical presentation**.

10-year cumulative incidence of non-target vessel revascularization in ACS versus CCS across subgroups based on age, sex, diabetes and stent generation. ACS indicates acute coronary syndrome; CCS, chronic coronary syndrome; CI, confidence interval; DES2G, newer-generation DES; HR, hazard ratio; pint, p value for interaction.

**SUPPLEMENTAL FIGURES**

**Figure S1**

**
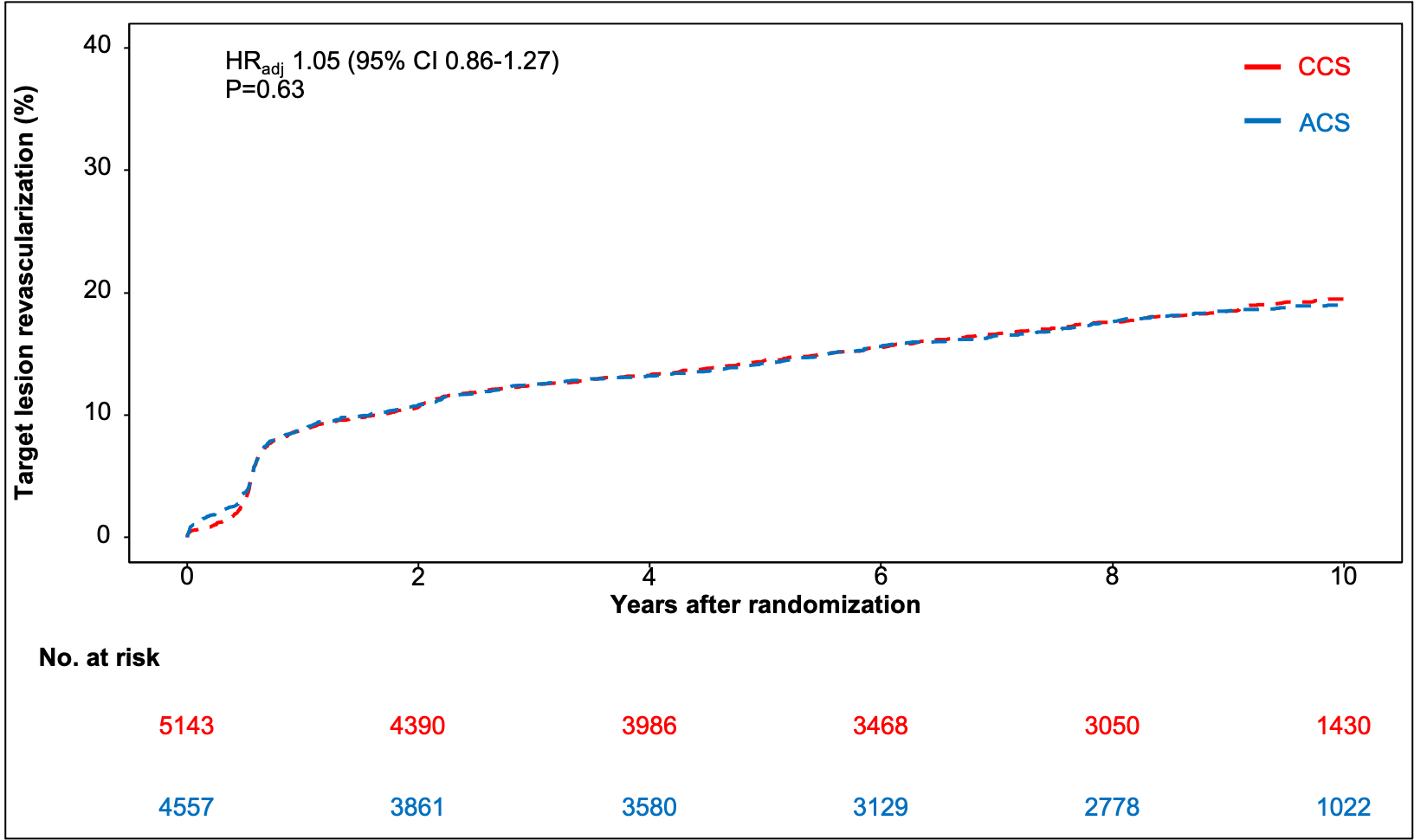
**

**Figure S2**

**
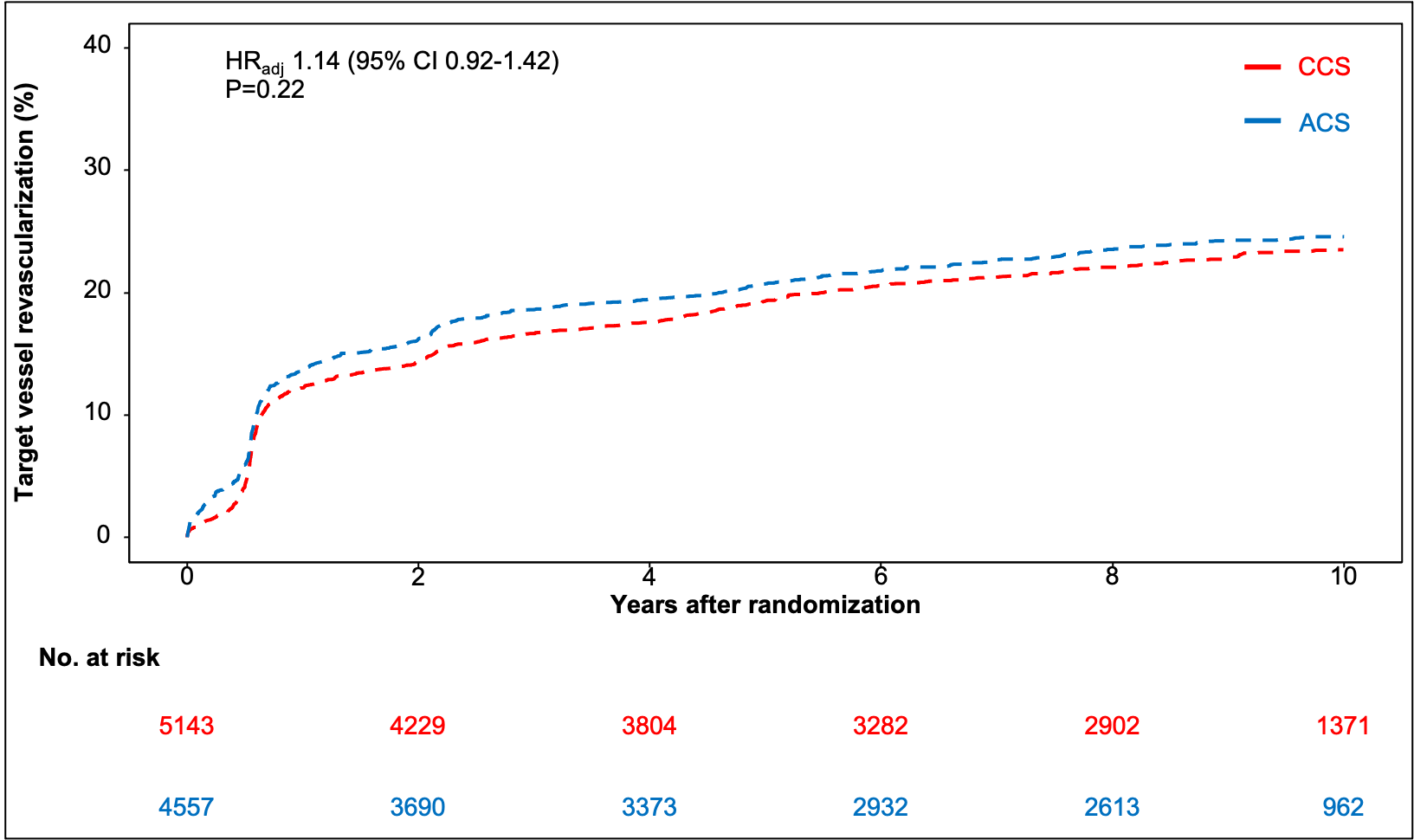
**

**Figure S3**

**
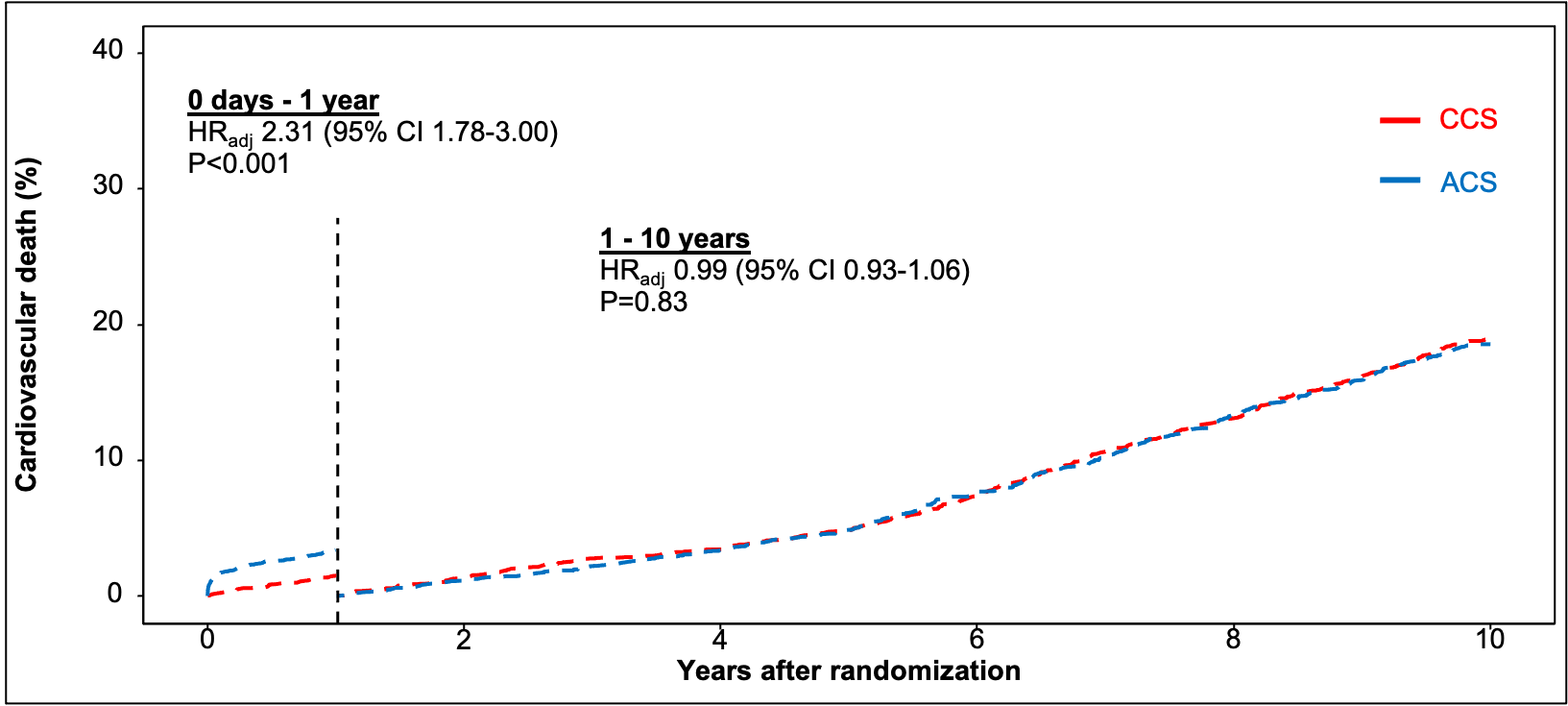
**

**Figure S4**

**
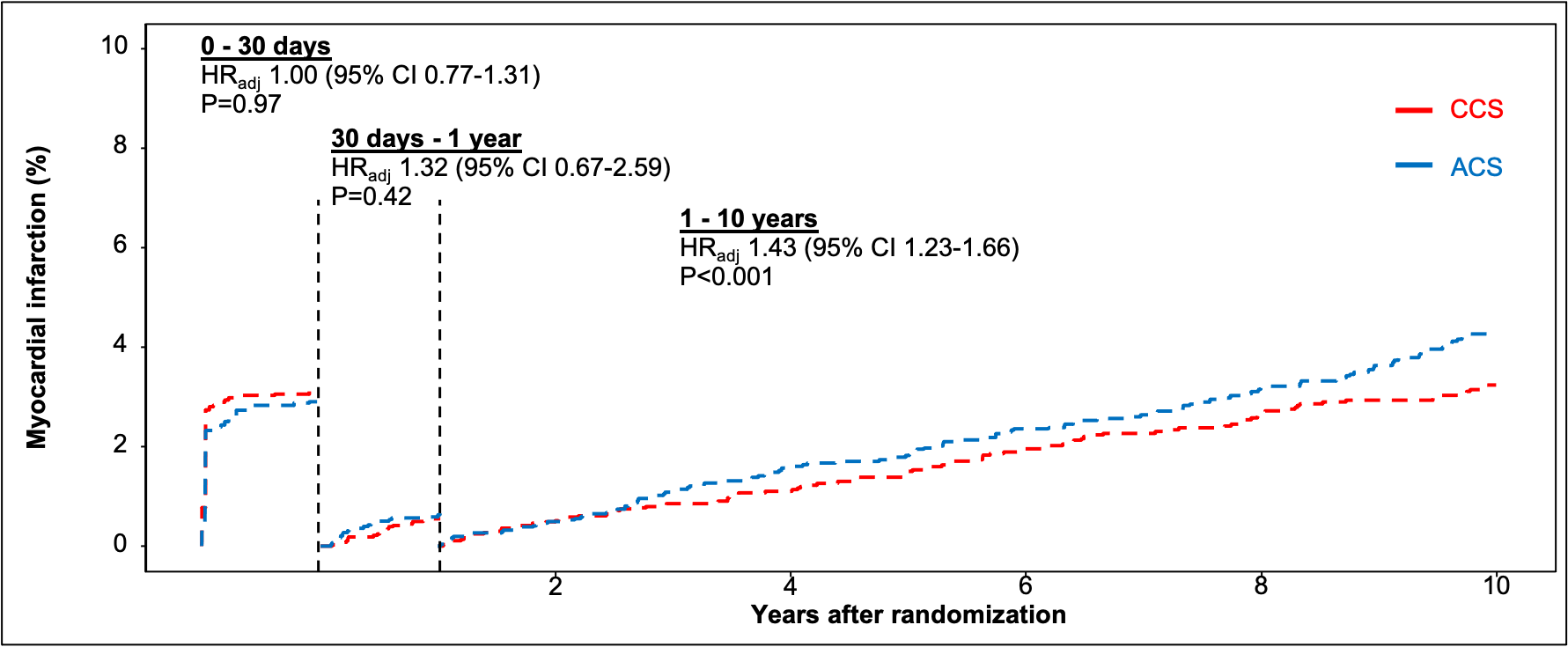
**

**Figure S5**

**
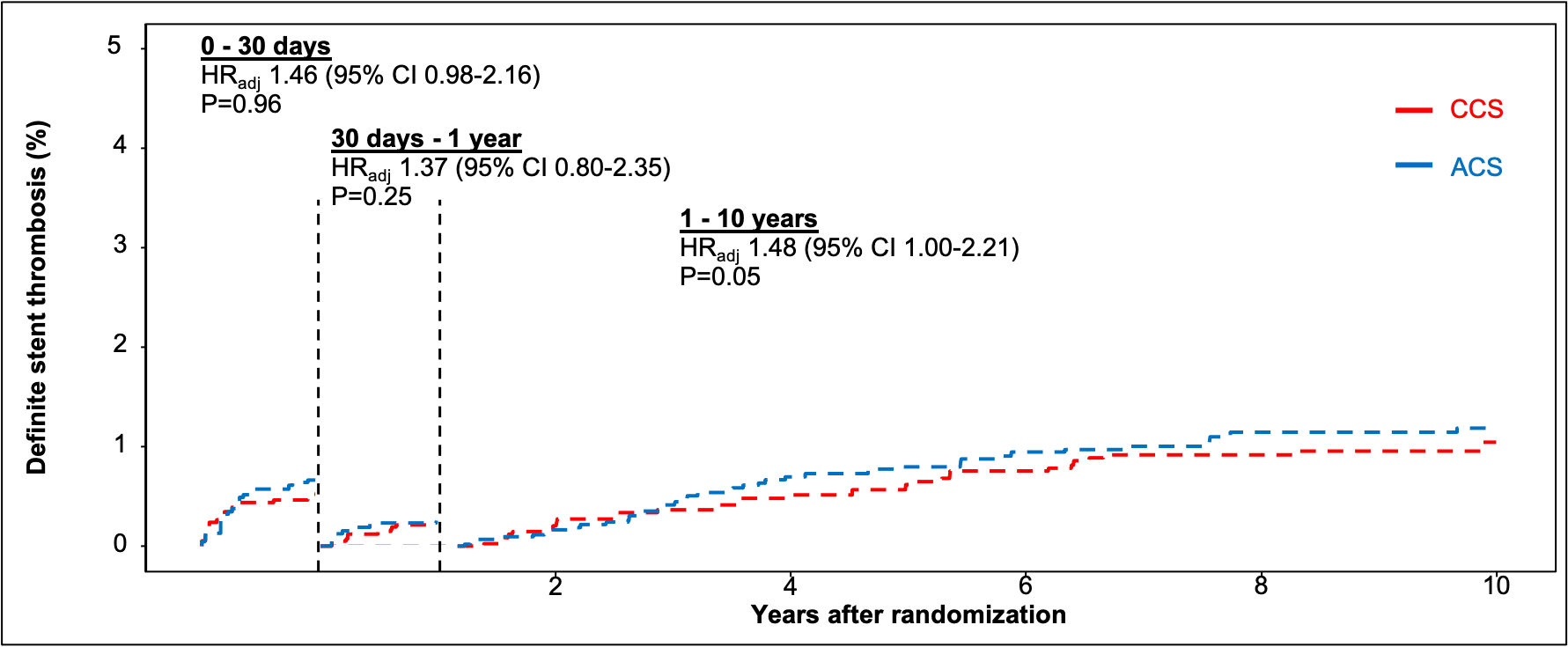
**

**Figure S6**

**
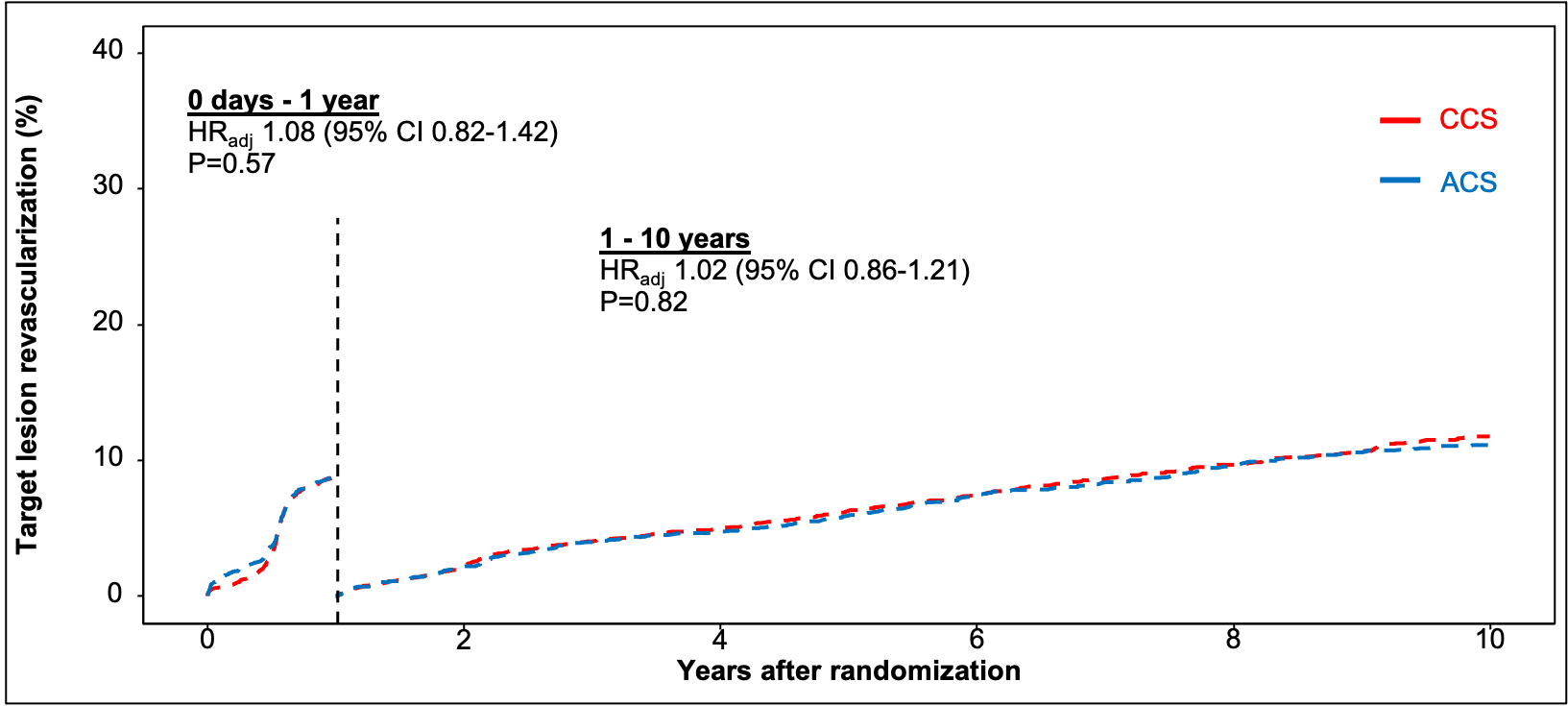
**

**Figure S7**

**
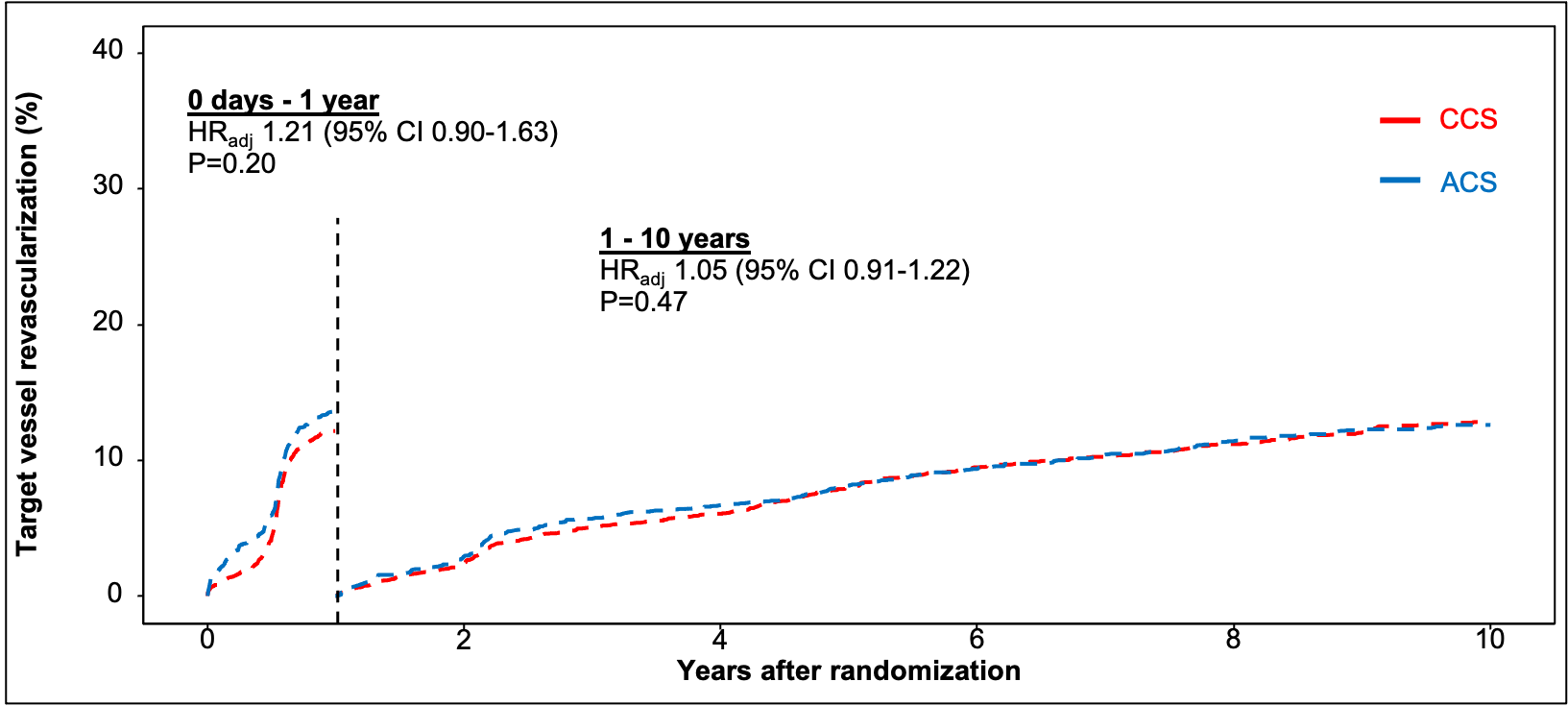
**

**Figure S8**

**
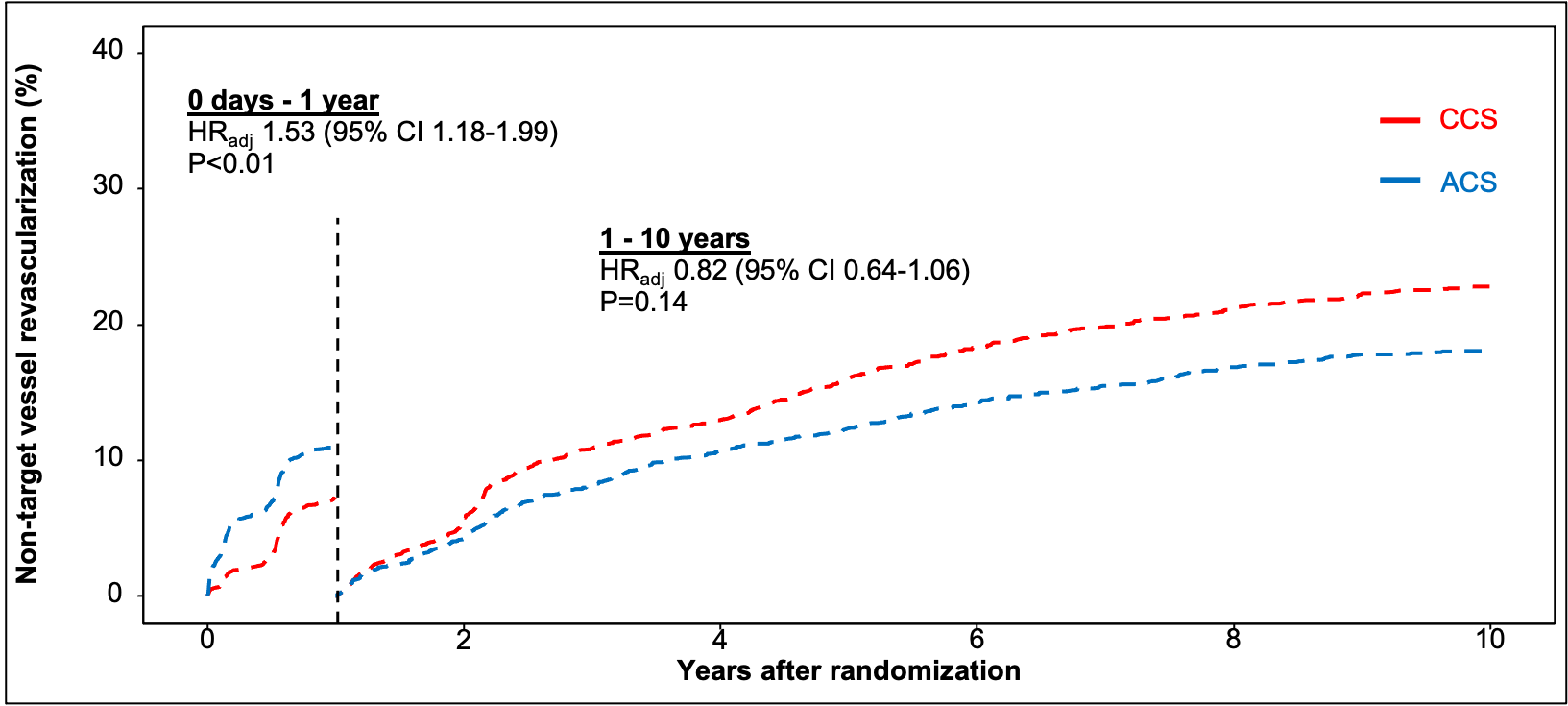
**

**Figure S9**


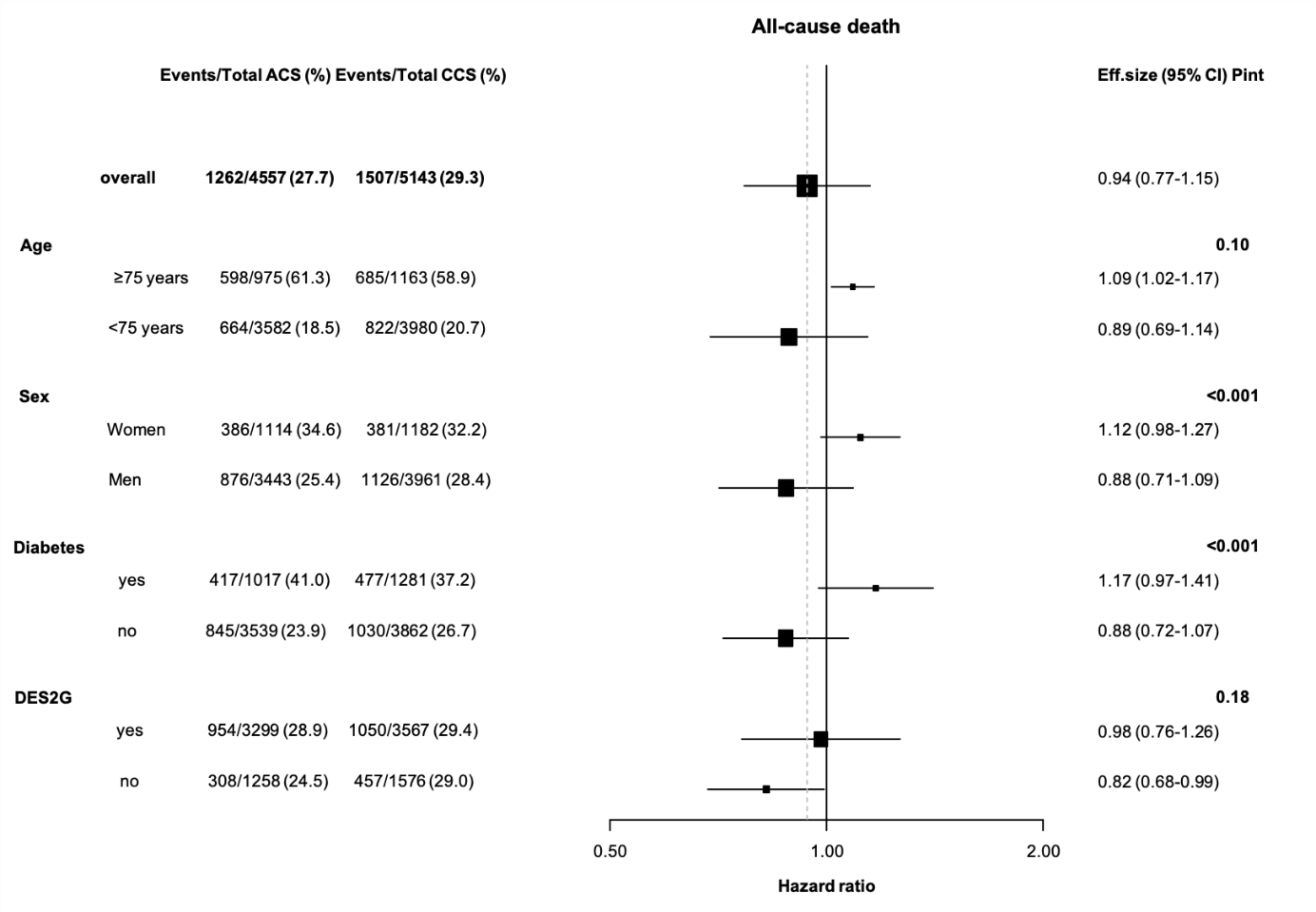


**Figure S10**


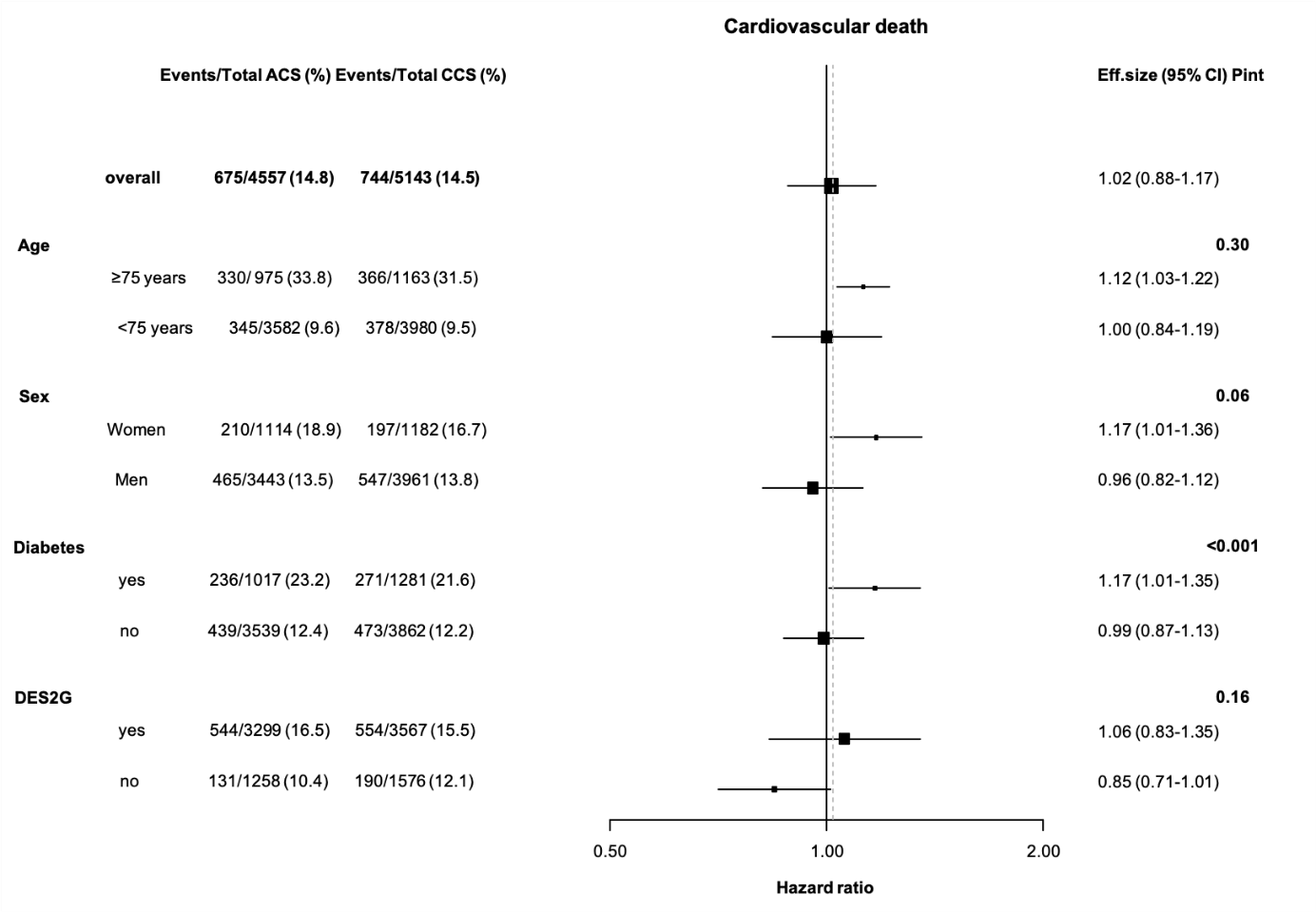


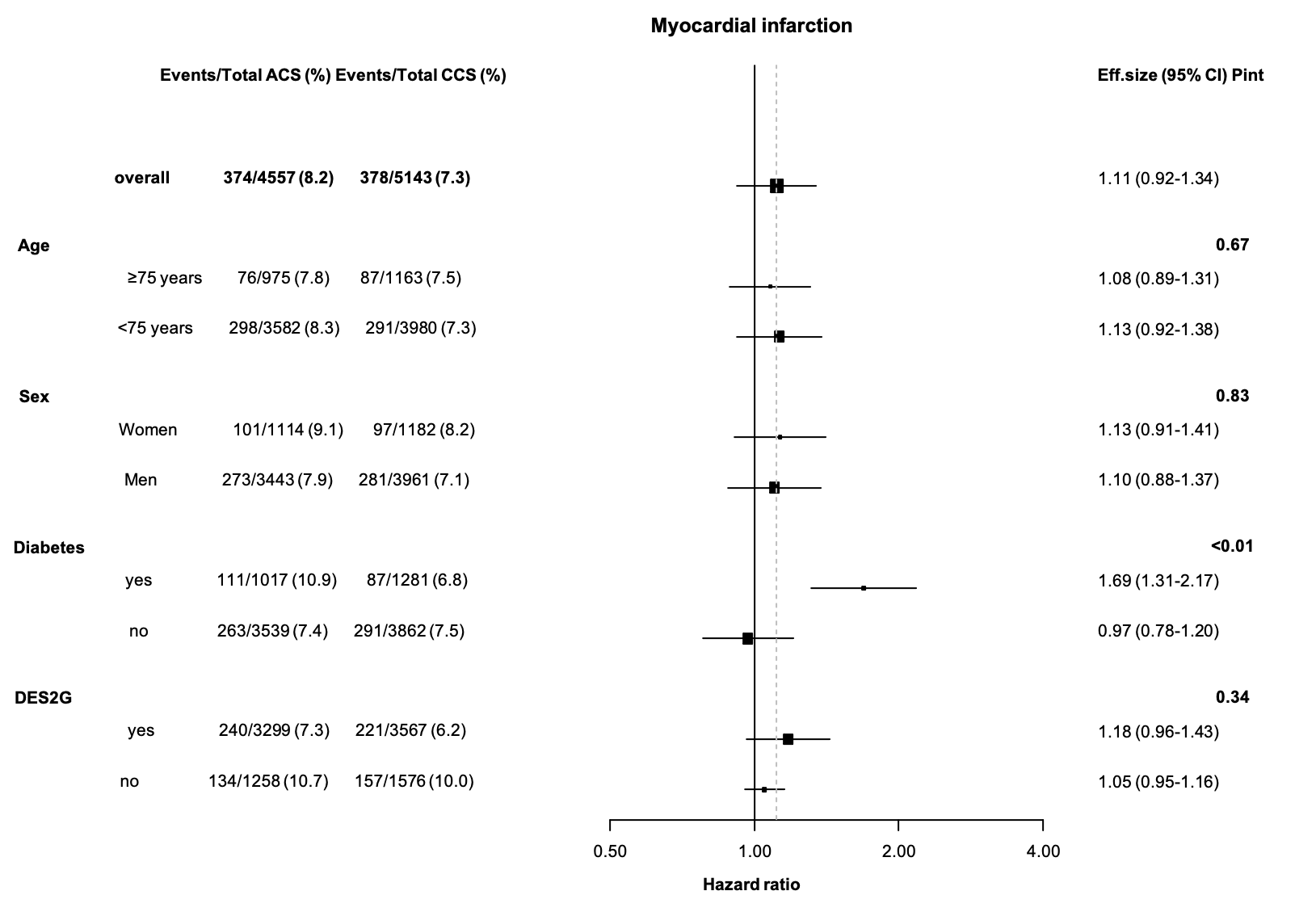
**Figure S11**

**Figure S12**


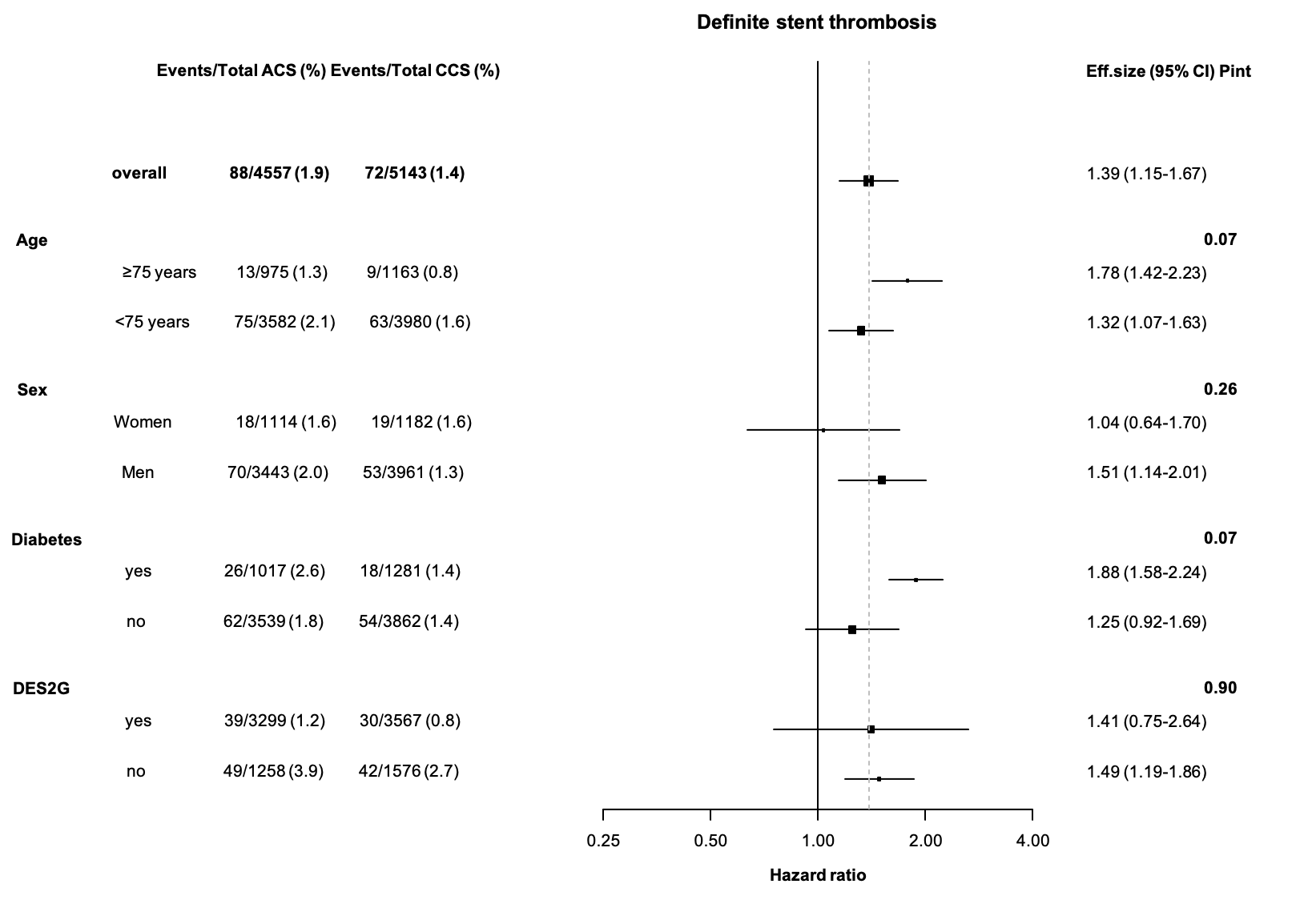


**Figure S13**


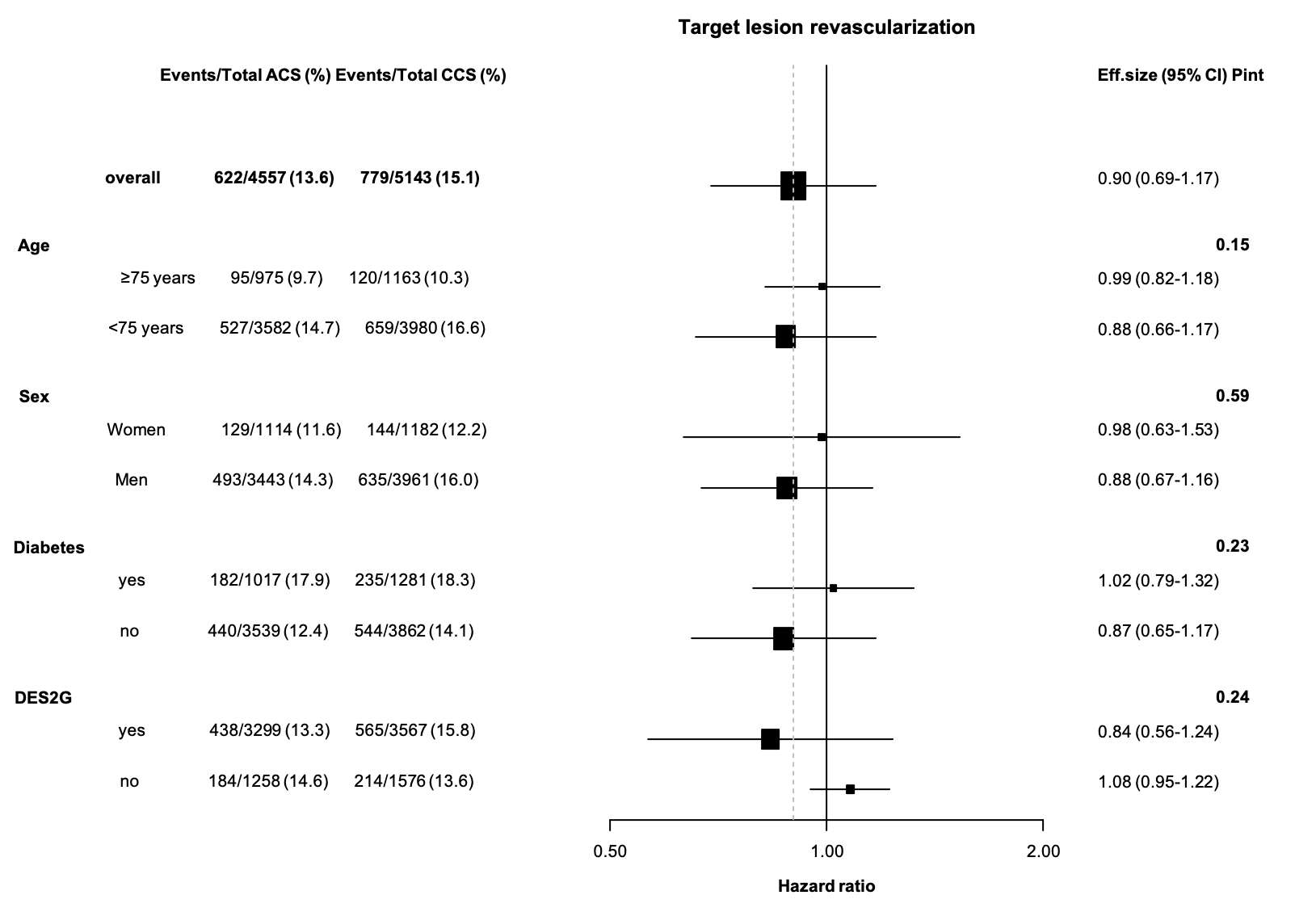


**Figure S14**


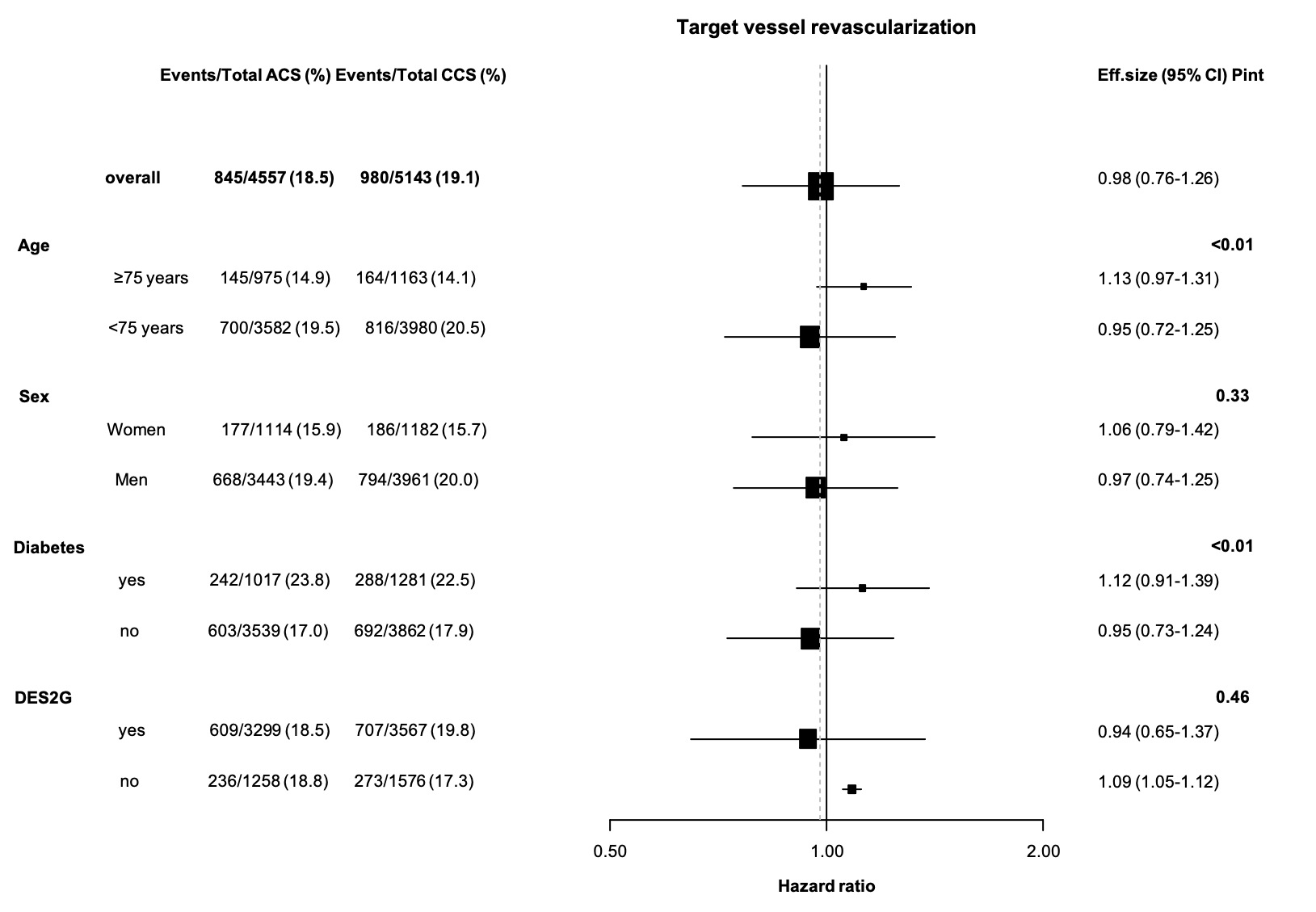


**Figure S15**


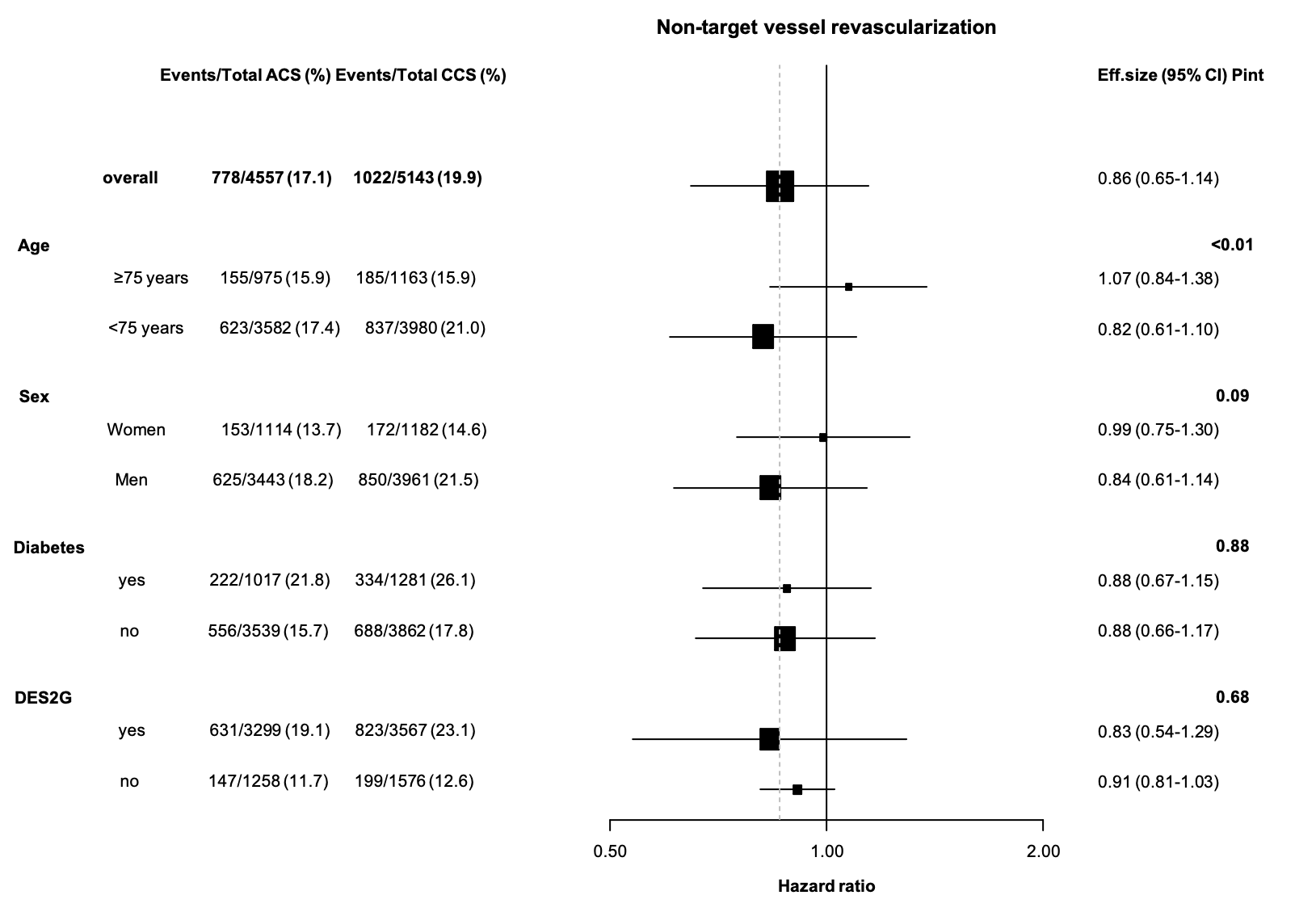

Supplement: Supplementary file 1 — Data S1. [file ECI-55-e14323-s001.docx]
